# Comprehensive protocols for culturing and molecular biological analysis of IBD patient-derived colon epithelial organoids

**DOI:** 10.3389/fimmu.2023.1097383

**Published:** 2023-02-23

**Authors:** Shreya Gopalakrishnan, Ingunn Bakke, Marianne Doré Hansen, Helene Kolstad Skovdahl, Atle van Beelen Granlund, Arne K. Sandvik, Torunn Bruland

**Affiliations:** ^1^ Department of Clinical and Molecular Medicine (IKOM), Faculty of Medicine and Health Sciences, NTNU- Norwegian University of Science and Technology, Trondheim, Norway; ^2^ Clinic of Laboratory Medicine, St. Olav’s University Hospital, Trondheim, Norway; ^3^ Centre of Molecular Inflammation Research (CEMIR), Faculty of Medicine and Health Sciences, NTNU- Norwegian University of Science and Technology, Trondheim, Norway; ^4^ Department of Gastroenterology and Hepatology, Clinic of Medicine, St. Olav’s University Hospital, Trondheim, Norway; ^5^ Clinic of Medicine, St. Olav’s University Hospital, Trondheim, Norway

**Keywords:** intestinal epithelium, intestinal epithelial organoids, colonoids, chemokines, gene expression, protein expression analysis, staining of paraffin sections, inflammatory bowel diseases

## Abstract

There are many unanswered questions regarding responses to proinflammatory signals in intestinal epithelial cells (IECs). For example, chemokines secreted by IECs upon external stimuli play multifunctional roles in both homeostasis and during inflammation. Several chemokines are upregulated during active inflammatory bowel disease (IBD), which is associated with an increased influx of immune cells into the gut mucosa. Therefore, studies on how chemokines are regulated in the intestinal epithelium may identify putative treatment targets in IBD. More recently, patient-derived *ex vivo* models such as intestinal organoids have facilitated molecular analysis of epithelial alterations in IBD patients own cells. Here, we describe refined experimental protocols and methods for the generation and maintenance of IBD patient-derived colonic organoids (colonoids) culture. We also give detailed description of medium, and supplements needed for colonoid establishment, growth, and differentiation, including production of Wnt-3A and Rspondin1 enriched media. Further, we present protocols for RNA and protein isolation from human colonoids, and subsequent gene expression analysis and Western blotting for e.g., signal transduction studies. We also describe how to process colonoids for chemokine protein expression analysis such as immunostaining, confocal imaging, and detection of secreted chemokines by e.g., enzyme-linked immunosorbent assay (ELISA). As proof of principle, we give examples of how the chemoattractant CCL20 can be regulated and expressed in colonoids derived from IBD-patients and healthy controls upon ligands-driven inflammation.

## Introduction

Inflammatory bowel disease (IBD) is a group of chronic inflammatory diseases of the gastrointestinal tract (1). Since IBD is primarily an inflammatory disease, most studies focus on the role of immune cells in the disease pathogenesis. However, more recently, the intestinal epithelial cells (IECs) are emerging as crucial immune modulators in IBD and have been identified as essential targets in IBD treatment (2). IECs are organized as crypts in the large intestine and crypts and villi in the small intestine, forming a monolayer consisting of several cell types such as stem-cells at the bottom of the crypts, and progenitor cells, absorptive- and secretory-differentiated cells further along the crypts and villi (3, 4). Together with mucus, this monolayer physically separates the underlying immune cells of the lamina propria from gut microbes. Moreover, the IECs and immune cells provide a chemical-immunological barrier by secreting mediators such as antimicrobial peptides, immunoglobulin A and cytokines (5). Upon microbial or proinflammatory stimuli IECs secrete chemokines - a class of chemoattractant cytokines that attract immune cells towards the gut mucosa (6). Several chemokines are upregulated in the mucosa of IBD patients who have increased infiltration of immune cells in the lamina propria (7). Chemokines can further induce the secretion of inflammatory cytokines that augment and prolong inflammatory responses during active inflammation in IBD (8, 9). IEC-derived chemokines are, therefore, potential targets for IBD treatment.

The regulation of chemokine expression and release in human IECs has been challenging to study due to lack of suitable model systems. More recently, patient-derived *ex vivo* models such as intestinal organoids have provided an excellent platform for translational research to understand better the specific pathways altered in individual IBD patients (10–15). Using methods developed by Sato et al. (16), crypts derived from biopsies of patients can be cultured in basement membrane matrix (Matrigel) along with medium containing factors critical for intestinal stem cell growth to re-establish themselves as three-dimensional spheroids that eventually form enteroids (in the small intestine) or colonoids (in the large intestine) (16, 17). Organoids accurately reproduce the functional epithelial mono-cellular layer, can be kept in long-term cultures and simulate *in vivo* environment by reacting to external stimuli to release e.g., chemokines into the media (i.e., conditioned media) (18–20). Thus, organoids retain *in vivo* epithelial monolayer characteristics, possibly even on a person-specific level, and allow detailed studies of IEC mechanisms relevant to e.g., inflammation.

**Figure 1:**
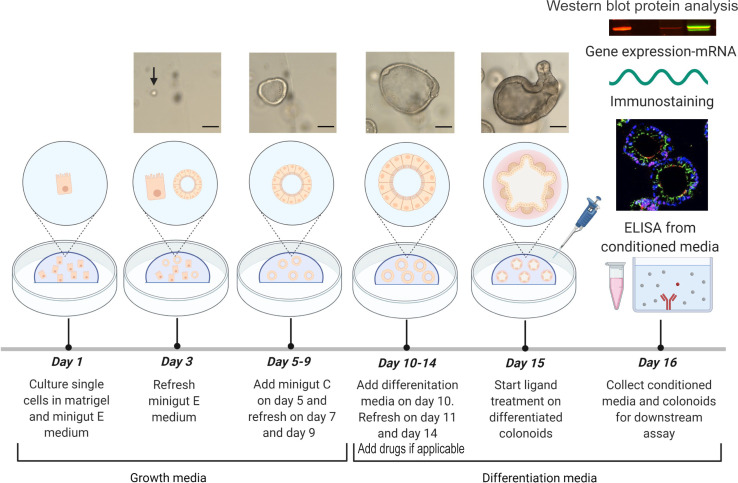
Experimental design. Colonoids are passaged and plated as single cells in Matrigel with minigut E media on day 1 and refreshed with minigut E media on day 3. Further, colonoids are given minigut C media from day 5 to day 9 with refreshment of media on alternate days (Protocol 4). On day 10, colonoids are subjected to differentiation media and refreshed with the same on day 11 and day 14 (Protocol 5). If doing drug experiments, drugs are included in the refreshed media on days 11 and 14- and one-hour pre ligand treatment. Ligand treatment is performed on day 15. Twenty-four hours post treatment with e.g., ligands, the conditioned media from colonoids are first collected (Protocol 5) before colonoids are harvested for downstream assays (Protocol 5) like immunostaining (Protocol 7), RNA isolation (Protocol 8) and Western blot (Protocol 9). Images of human colonoids taken on different days of culture from single cells are illustrated. Upon differentiation of colonoids on day 10, colonoids form three-dimensional multi-cell structures containing a central lumen and crypt-like protrusions containing different colonic cell types as seen on day 15. Images of colonoids represented in the illustration are taken at 10x objective with EVOS™ FL Auto 2 Imaging System (Thermo Fisher Scientific) and scale bars represent 100 µm. The illustration of the experimental design was created with BioRender.com.

In this protocol we first present an updated overview of how to generate human colonoids from crypts isolated from fresh and frozen biopsies. We then describe an experimental workflow for differentiation of colonoids, treatment with IBD relevant pro-inflammatory ligands and drugs, and subsequent collection of conditioned media and cells for downstream assays (**Figure 1**). We present refined protocols for RNA and protein isolation for gene expression and intracellular signaling by western blotting, respectively. We describe how to prepare colonoids for detection of e.g., chemokines by immunostaining and immunofluorescence, and how to measure chemokine release upon pro-inflammatory stimulation of IBD patient-derived colonoids. We have also included a section with tips and troubleshooting based on our experience with this methodology over the years that we hope will be helpful for researcher who wants to use patient-derived colonoids to study e.g., pro-inflammatory responses in human intestinal epithelium *ex vivo.*

## Methods and protocols

### Materials and methods

Materials, including preparation of buffers and media, are presented in **Tables 1**–**8**.

### Protocol 1 Production of Wnt-3A enriched media (minigut A)

All equipment, materials, reagents, and prepared buffers needed for these procedures are listed in **Table 1**. We add recombinant human Noggin (#120-10c Peprotech) to combined batches of Wnt-3A and Rspondin1 enriched media and adjust media compositions to fit our experimental approaches. Wnt-3A and Rspondin1 enriched media (see Protocol 2) are generated separately from the two cell lines L-Wnt-3A CRL-2647™ (RRID : CVCL 0635) and # AMS. RSPO1-CELLS (RRID : CVCL_RU08), respectively (**Table 1**). The cell line L-WRN-CRL ATCC (Cat #CRL-3276; RRID : CVCL_DA06) is an alternative source for producing Wnt-3A, R-spondin3, and Noggin into a single conditioned medium (WNR CM). Protocols for WNR CM and proven support of maintenance and propagation of stem cells in human colonoids are presented elsewhere (21, 22). Below is a protocol for production of Wnt-3A enriched media (minigut A).

1. On day 1, pre-warm complete growth media in a water bath at 37°C and sterilize the laminar flow hood with 70% EtOH. Always sterilize tools and plasticware with 70% EtOH before moving it into the hood.
2. Thaw the frozen vial of L-Wnt-3A CRL-2647™ cells rapidly in a water bath at 37 °C (can take up to two minutes). Once thawed, move the cells to sterile laminar flow hood.
3. Transfer the cells from the vial to a 15 mL centrifuge tube containing 9 mL complete growth media at room temperature and spin at 125 x *g* for 7 minutes at 4 °C.
4. Aspirate and discard supernatant (*see* note 1). Resuspend cells with 10 mL complete growth media, plate cells in a T75 cm^2^ flask, and transfer the flask to the incubator.
5. Monitor the growth of cells every day under a microscope until confluency.
6. On day 5, pre-warm growth media and complete growth media in a water bath at 37 °C and sterilize the laminar flow hood with 70% EtOH. Passage the cells by removing growth media, washing with 5 mL of DPBS, and trypsinizing with 1 mL Trypsin-EDTA for 10 minutes at 37 °C in the incubator (*see* note 2). Once cells are trypsinized, take 500 µL of cell suspension into a 50 mL tube containing 47 mL of pre-warmed complete growth media and triturate with a 10 mL pipette to dissociate the cells. Transfer the cells in growth media equally to two T175 cm^2^ flasks. Place the two T175 cm^2^ flasks in the incubator and follow the growth for the next 4 days, until cells are confluent.
7. On day 9, when the two T175 cm^2^ flasks are confluent, wash the flasks with 10 mL DPBS and trypsinize cells with 2 mL trypsin-EDTA per T175 cm^2^ flask. Once the cells are trypsinized, add 8 mL of growth media per flask to stop trypsinization and collect them in a 50 mL tube and triturate cells with 10 mL pipette several times to dissociate the cells. Centrifuge at 400 x *g* for 10 minutes at 4 °C.
8. Aspirate the supernatant and add 16 mL of pre-warmed growth media to the cells and triturate well with a 10 mL pipette. Thus, we harvest the cells when the flasks are confluent and resuspend the cells in a fixed volume (i.e 16 ml). Then, 1-5 mL cell suspensions are used independent of cell numbers, as described below:
9. Take 1 mL of cell suspension into T175 cm^2^ flask containing 22.5 mL of complete growth media with 0.8% geneticin. Repeat the same for another T175 cm^2^ flask. Label these flasks as A and B. Transfer the flasks to the incubator.
10. Take 1 mL of cell suspension into T175 cm^2^ flask containing 22.5 mL of growth media without geneticin. Repeat the same for another T175 cm^2^ flask. Label these flasks as C and D. Transfer the flasks to the incubator.
11. Take 5 mL of cell suspension into a multi-layer cell culture flask containing 112.5 mL of growth media without geneticin. Repeat the same with another multi-layer cell culture flask. Label these flasks as E and F (*see* note 3). Transfer the flasks to the incubator.
12. On day 13, aspirate media from flasks C, D, E and F and transfer the media to 50 mL centrifuge tubes (*see* note 3). Refresh flasks C and D with 23.5 mL per flask and flasks E and F with 117.5 mL per flask of freshly prepared growth media without geneticin, pre-warmed at 37 °C. Place the flasks back in the incubator. On day 13, flasks A and B must be split for producing a new batch of media. See step 18 for the procedure (also *see* note 4).
13. Centrifuge the 50 mL tubes containing the media collected from flasks C, D, E and F at 600 x *g* for 10 minutes at 4 °C. Aspirate supernatant from all the 50 mL tubes to a 500 mL glass bottle and freeze at -20 °C. Label this bottle as Wnt-3A1.
14. On day 16, place the glass bottle containing Wnt-3A1 at 4 °C to defrost overnight. On day 17, place the Wnt-3A1 at room temperature if the Wnt-3A1 was not defrosted overnight at 4°C.
15. On day 17, aspirate the growth media without geneticin added on day 13 to flasks C, D, E and F (step 12). Process them as described in step 13. Label this batch of media as Wnt-3A2.
16. Prepare minigut A (Wnt-3A enriched media) as described in **Table 1**.
17. Filter minigut A through a media bottle filtration unit, aliquot 40 mL of minigut A into 50 mL tubes inside laminar flow hood and freeze at -20°C until used for colonoid cultures.
18. The two T175 cm^2^ flask labelled as A and B containing complete growth media (with geneticin, step 9) can be similarly passaged and further grown without geneticin (follow steps 7 to 17) to obtain more Wnt-3A media (*see* note 4). We preserve minigut A media for 6-8 months in the freezer.
19. The Wnt and Rspondin activity (see Protocol 2) can be monitored using TCF/LEF-luc-reporter cell line (BPS Bioscience #60501). The activity is measured by Luciferase assay from Promega as per manufacturer’s instruction (Promega #E1500). See Results and Discussion for further considerations.

**Table 1:** Production of Wnt-3A enriched media (minigut A) and Rspondin1 enriched media.

Equipment			
Two autoclaved glass bottles with lid, 500 mL		Cell culture flasks, T75 cm^2^ and T175 cm^2^	
Laminar flow hood		Pipettes, 10 mL and 25 mL	
Water bath at 37 °C		Sterile centrifuge tubes, 15 mL and 50 mL	
Incubator at 37 °C with 5% CO_2_		Pipettes and filter tips, P200 and P1000	
Centrifuge at 4 °C for 15 mL and 50 mL tubes			
Inverted microscope for cell cultures			
Materials and reagents				Name		Catalog number	Manufacturer
Ethanol (EtOH) 70%			
Dimethyl sulfoxide (DMSO)			
Fetal calf serum (FCS), heat inactivated			
Media bottle filtration unit		514-0297	VWR
Multi-layer cell culture flasks 875 cm^2^		734-2457	VWR
HEPES (1M)		15630106	Thermo Fisher Scientific
Bovine serum albumin (BSA)		A7906-500G	Sigma-Aldrich
Trypsin-Ethylenediaminetetraacetic acid (EDTA)		BE17-161E	Lonza
*Zeocin (100 mg/mL)		R25001	Invitrogen
Dulbecco’s Modified Eagle Medium (DMEM), high glucose, GlutaMAX supplement, pyruvate		31966	Thermo Fisher Scientific
*Advanced DMEM/F12		12634(028)	Thermo Fisher Scientific
Dulbecco’s phosphate buffered saline without Ca^2+^ and Mg^2+^ (DPBS), sterile		D8537-500ML	Merck
Gibco penicillin-streptomycin (10,000 U/mL)		11548876	Thermo Fisher Scientific
Geneticin Selective Antibiotic (G418 Sulfate) (50 mg/mL)		10131027	Thermo Fisher Scientific
GlutaMAX Supplement (100x)		35050061	Thermo Fisher Scientific
B27 Supplement, serum free (50x)		17504001	Thermo Fisher Scientific
N-2 Supplement (100x)		17502001	Thermo Fisher Scientific
^§^L-Wnt-3A producing cells		L-Wnt-3A CRL-2647™ (RRID : CVCL_0635)	ATCC^®^
* Rspondin1 producing 293-HA-Rspo1-Fc cells		AMS. RSPO1-CELLS (RRID : CVCL_RU08)	AMS Biotechnology, Abington, United Kingdom
Prepared buffers				Name	Preparation		
^§^Growth medium for L-Wnt-3A cell line	DMEM, high glucose, GlutaMAX, pyruvate with 10% (v/v) FCS.		
^§^Complete growth medium for L-Wnt-3A cell line	DMEM, high glucose, GlutaMAX, pyruvate with 10% (v/v) FCS and 0.8% (v/v) Geneticin Selective Antibiotic.		
*Growth medium for Rspondin1 cell line	DMEM, high glucose, GlutaMAX, pyruvate with 10% (v/v) FCS and 100 U/mL penicillin-streptomycin.		
*Complete growth medium for Rspondin1 cell line	DMEM, high glucose, GlutaMAX, pyruvate with 10% (v/v) FCS, 100 U/mL penicillin-streptomycin, and 300 µg/mL Zeocin.		
*AD-DF++ medium for Rspondin1 cell line	Advanced DMEM/F12 with 10 mM HEPES, 1x GlutaMAX and 100 U/mL penicillin-streptomycin.		
^§^Wnt-3A enriched media (minigut A)	Mix equal volumes of Wnt-3A1 and Wnt-3A2 as described in Protocol 1. Add to a final concentration; BSA 1% (w/v), GlutaMAX 1% (v/v), HEPES 1% (10 mM), Penicillin-streptomycin 1% (100 U/mL), N-2 Supplement 1% (1x) and B27 Supplement 2% (1x).		
*Rspondin1 enriched media	Prepare from supernatant obtained from two multi-flasks per batch as described in Protocol 2. Add to a final concentration; BSA 1% (w/v), GlutaMAX 1% (v/v), HEPES 1% (10 mM), Penicillin-streptomycin 1% (100 U/mL), N-2 Supplement 1% (1x) and B27 Supplement 2% (1x).		

*Only needed for production of Rspondin1 enriched media. ^§^Only needed for production of Wnt-3A enriched media.

### Protocol 2 Production of Rspondin1 enriched media

1. Always sterilize tools and plasticware with 70% EtOH before moving it into the hood. Follow steps 1-7 from Protocol 1 (also *see* notes 1-4) for growing the 293-HA-Rspol-Fc cells. However, use the complete growth medium (with zeocin and penicillin-streptomycin) and growth medium (with penicillin-streptomycin) for the Rspondin1 cell line instead of the media for the Wnt-3A cell line (**Table 1**).
2. Post-centrifugation on day 9 aspirate the supernatant and add 14 mL of pre-warmed growth media and triturate well with a 10 mL pipette.
3. Take 1 mL of cell suspension into T175 cm^2^ flask containing 22.5 mL of complete growth media (with zeocin). Repeat the same for another T175 cm^2^ flask. Label these flasks as A and B. Transfer the flasks to the incubator.
4. Take 5 mL of cell suspension into a multi-layer cell culture flask containing 112.5 mL of growth media (without zeocin). Repeat the same with another multi-layer cell culture flask. Label these flasks as C and D. Transfer the flasks to the incubator.
5. On day 13, when the multi-layer flasks C and D are confluent (*see* note 5), aspirate the growth media from the multi-layer cell culture flasks and discard the media. Refresh multi-layer cell culture flasks with 250 mL of AD-DF++ media per flask and place them in the incubator for one week.
6. On day 13, flasks A and B have to be split for producing a new batch of media as described in step 9.
7. On day 20, collect the AD-DF++ media from the multi-layer flasks C and D into 50 mL tubes and spin them at 600 x *g* for 10 minutes at 4°C (*see* note 6). Aspirate supernatant and prepare 500 mL of Rspondin1 enriched media obtained from two multi-flasks per batch as described in **Table 1**.
8. Filter Rspondin1 enriched medium through media bottle filtration unit, aliquot 20 mL volumes into 50 mL tubes and freeze at -20°C until used for colonoid cultures.
9. The two T175 cm^2^ flask labelled as A and B containing complete growth media (with zeocin) can be similarly passaged and further grown without zeocin for 4 days followed by addition of AD-DF++ media (steps 1 to 8) to obtain another batch of media (*see* notes 4-6). We preserve Rspondin1 enriched media for 6-8 months in the freezer.

### Protocol 3 Isolation of crypts from fresh and frozen colonic biopsies

Colonic crypts can be isolated directly from fresh pinch biopsies collected during colonoscopy or the biopsies can be stored in freezing media in a liquid nitrogen tank for later crypt isolation. We usually isolate crypts from 4-5 biopsies per donor. All equipment, materials, reagents, and prepared buffers needed for these procedures are listed in **Tables 2**, **3**.

1. Always sterilize tools and plasticware with 70% EtOH before moving it into the hood. Place a 24-well plate with lid in the incubator. Sterilize the laminar flow hood with 70% EtOH. Thaw Matrigel by placing the tubes on ice for 3-4 hours. Keep DPBS and chelation buffer ready on ice.
2. Collect the biopsies into 25 mL ice-cold DPBS, bring them to sterile laminar flow hood in a container with ice. Biopsies in DPBS should always be kept on ice.
3. Take a 10 mL pipette and wash the biopsies by passing up and down into the pipette, let the biopsies sink to the bottom of the tube, and discard the supernatant. Repeat up to 5 times by adding fresh ice-cold DPBS each time or until the supernatant is clear (*see* note 7).
4. If performing crypt isolation, proceed with step 6.
5. If freezing down the biopsy, use the following protocol (23):
  - Fill 1 mL of freezing media in the cryotubes. Place one biopsy per mL of freezing media in the cryotubes.
  - Transfer the cryotubes to Mr. Frosty freezing container and store them at -80 °C for 48 hours. Transfer the frozen biopsies from -80 °C to the liquid nitrogen tank for longer storage.
  - When taking up the frozen biopsies from liquid nitrogen tank for crypt isolation, rapidly thaw the freezing media with the biopsies in a water bath at 37 °C, until there is a small amount of the frozen media left in the tube. Take the tube into the laminar flow hood and carefully aspirate the freezing media and discard it.
  - Wash biopsies with 1 mL of Advanced DMEM/F12 containing 10% FCS at room temperature. Aspirate media and gently wash the biopsies with ice-cold DPBS 3-4 times to remove the DMSO from the biopsies.
6. Place the biopsies on a silicone-coated petri dish filled with 40 mL ice-cold DPBS.
7. Place the petri dish with biopsies under a microscope. Using insect pins and curved forceps, stretch the biopsies and pin them with the mucosal side facing upwards (*see* note 8). Scrape gently to remove mucus. Work quickly to avoid warming of the DPBS.
8. Bring the petri dish with biopsies back into the laminar flow hood and place the petri dish on top of ice in an icebox. Remove the DPBS containing debris. Add 20 mL of ice-cold chelation buffer and gently swirl the petri dish to wash off any excess debris and discard the chelation buffer with debris. Repeat this step 3-4 times.
9. Add 40 mL of ice-cold 2 mM EDTA chelation buffer to completely cover all the biopsies pinned to the petri dish.
10. Cover the petri dish with a lid and add plenty of ice in the icebox. Place the icebox on an orbital shaker with gentle shaking at 200 rpm for 30 minutes.
11. Bring the petri dish with the biopsies back into the laminar flow hood and discard the EDTA chelation buffer carefully without disturbing the biopsies (*see* note 9).
12. Carefully add 40 mL of ice-cold chelation buffer without EDTA. Swirl the petri dish once or twice and discard the supernatant. Repeat this step 2 more times (*see* note 9).
13. Refresh petri dish once more with ice-cold chelation buffer without EDTA and take the petri dish under a light microscope.
14. Scrape the mucosa with curved forceps and remove all crypts from the biopsies. Due to incubation with chelation buffer containing EDTA in the previous step, scraping will release the crypts. Crypts released from the biopsies will be visible in the chelation buffer under the microscope.
15. Bring the petri dish back into the laminar hood. Keep a new 50 mL tube ready on ice. Gently, without triturating many times, aspirate the chelation buffer from the petri dish (containing the crypts) using a 10 mL pipette and transfer them to the 50 mL tube placed on ice.
16. Filter the chelation buffer containing crypts through 150 µm nylon filter mesh to remove excess debris from the suspension and collect the crypt fraction into a new 50 mL tube placed on ice (*see* note 10).
17. Centrifuge the crypts at 50 x *g* for 5 minutes at 4°C. Carefully discard the supernatant (*see* note 11).
18. Resuspend the crypt pellet with 1 mL of ice-cold chelation buffer. Transfer the suspension to a sterile 1.5 mL microfuge tube and centrifuge the tube at 200 x *g* for 10 minutes at 4°C. Aspirate supernatant carefully and place pellet on ice.
19. Mix the thawed Matrigel with a P1000 pipette (*see* note 12) and add 300 µL of Matrigel to the pellet with crypts. Mix thoroughly without creating air bubbles and place tube back on ice.
20. Transfer the 24-well plate from the incubator into the laminar flow hood. Using P200 pipette, pipette 50 µL of the Matrigel with crypts per well (*see* notes 12 and 13).
21. Place the 24-well plate in the incubator for 20 minutes. Add 500 µL of minigut D to the side of all the wells carefully without disturbing the Matrigel with crypts. After 48 hours, aspirate minigut D and add minigut C. Further, refresh with minigut C growth media every 2 days until the colonoids are ready for passaging.

**Table 2:** Materials for isolation of crypts from fresh and frozen colonic biopsies.

Equipment		
Two stainless steel curved forceps	Sterile 24-well flat bottom cell culture plates with lid	
Light microscope, Nikon SMZ-2B	Pipette, 10 mL	
Orbital shaker	Sterile microfuge/centrifuge tubes, 1.5 mL and 50 mL	
Silicone coated glass petri dish with lid	*Cryotubes, 2mL	
Laminar flow hood	Pipettes and filter tips, P200 and P1000	
*Water bath at 37 °C	0.22 µm sterile filter	
Incubator at 37 °C with 5% CO_2_	150 µm nylon filter mesh	
Centrifuge at 4 °C for 1.5 mL and 50mL tubes	Ice and icebox	
Freezers -20 °C and -80 °C	*Liquid nitrogen	
Materials and reagents		
Name	Catalog number	Manufacturer
Six colonic pinch biopsies taken with Olympus FB-240K forceps		
Sorbitol		
Sucrose		
EtOH 70%		
DMSO		
FCS, heat inactivated		
*Mr. Frosty freezing container, pre-filled with isopropanol until the marked line in the box	5100-001	Thermo Fisher Scientific
Minutien Insect Pins 0.1 mm Stainless steel	26002-10	Fine Science Tools
Media bottle filtration unit	514-0297	VWR
DPBS, sterile and ice-cold	D8537-500ML	Merck
Ultrapure EDTA (0.5M)	15575-038	Invitrogen
BSA	A7906-500G	Sigma-Aldrich
HEPES (1M)	15630106	Thermo Fisher Scientific
Gibco penicillin-streptomycin (10,000 U/mL)	11548876	Thermo Fisher Scientific
B27 Supplement, serum free (50x)	17504001	Thermo Fisher Scientific
N-2 Supplement (100x)	17502001	Thermo Fisher Scientific
Gentamicin/Amphotericin solution (500x)	R01510	Thermo Fisher Scientific
GlutaMAX Supplement (100x)	35050061	Thermo Fisher Scientific
*Advanced DMEM/F12 with 10% (v/v) FCS	12634(028)	Thermo Fisher Scientific
Matrigel^®^ Growth Factor Reduced (GFR) – Freeze 1 ml aliquots at -20 °C until use (*see* Note 12)	CLS354230-1EA	Merck (Corning^®^)
CHIR99021 - Prepare 4.65 mg/mL stock in DMSO, freeze 10 µL aliquots at -20 °C until use	72052	STEMCELL Technologies
Thiazovivin - Prepare 3.11 mg/mL stock in DMSO, freeze 10 µL aliquots at -20 °C until use (Note 18).	72252	STEMCELL Technologies
Nicotinamide - Prepare 122.12 mg/mL stock in DPBS, freeze 500 µL aliquots at -20 °C until use	N3376-100G	Merck
N-Acetyl-L-cysteine - Prepare 163.19 mg/mL stock in DPBS, freeze 50 µL aliquots at -20 °C until use	A9165-25G	Sigma-Aldrich
A-83-01 - Prepare 0.21 mg/mL stock in DMSO, freeze 50 µL aliquots at -20 °C until use	SML0788	Sigma-Aldrich
[Leu]15-Gastrin 1 - Prepare 0.21 mg/mL stock in DPBS, freeze 10 µL aliquots and at -20 °C until use.	G9145-1MG	Sigma-Aldrich
SB202190 - Prepare 9.94 mg/mL stock in DMSO, freeze 34 µL aliquots at -20 °C until use	S7067	Sigma-Aldrich
Human EGF - Prepare 0.5 mg/mL stock in DPBS, freeze 10 µL aliquots at -20 °C until use	AF-100-15	Peprotech
Human Noggin - Prepare 0.1 mg/mL stock in DPBS, freeze 50 µL aliquots at -20 °C until use	120-10c	Peprotech

*Only needed if doing crypt isolation from frozen colonic biopsies.

**Table 3:** Prepared buffers for isolation of crypts from fresh and frozen colonic biopsies.

Prepared buffers	
Name	Preparation
*Freezing media for biopsy	Advanced DMEM/F12 with 10% (v/v) DMSO, and 20% (v/v) FCS
Wnt-3A enriched media (minigut A)	In-house, *see* Protocol 1, notes 1-4 and **Table 1**
Rspondin1 enriched media	In-house, *see* Protocol 2, notes 1-6 and **Table 1**
Minigut B	Advanced DMEM/F12 with 1% (w/v) BSA, 1x GlutaMAX, 10 mM HEPES, 100 U/mL penicillin-streptomycin, 1x N-2 supplement and 1x B27 supplement. Minigut B is prepared in 2 L volume, sterile filtered with 0.22 µm filter, aliquoted as 30 mL aliquots in 50 mL tubes and stored at -20 °C until use.
Chelation buffer	Sterile DPBS with 2% (w/v) sorbitol, 1% (w/v) sucrose, 1% (w/v) BSA and 0.2% (v/v) Gentamicin/Amphotericin solution (10 µg/mL Gentamicin and 0.25 µg/mL Amphotericin B final concentration). Prepare fresh, sterile filter with 0.22 µm filter and freeze in 40 mL aliquots.
EDTA (2 mM) chelation buffer	Chelation buffer with 0.4% of 0.5M EDTA stock.
Establishment media (minigut D)	50% (v/v) minigut A, 30% (v/v) minigut B, 20% (v/v) Rspondin1 enriched media, 1221.2 µg/mL nicotinamide, 163.19 µg/mL N-Acetyl-L-cysteine, 0.1 µg/mL human Noggin, 0.211 µg/mL A-83-01, 3.314 µg/mL SB202190, 0.05 µg/mL Human EGF, 0.021 µg/mL [Leu]15-Gastrin 1, 1.163 µg/mL CHIR99021 and 0.778 µg/mL Thiazovivin (see Note 18). Prepare fresh and sterile filter with 0.22 µm filter. Pre-warm at 37 °C for 15 minutes before use.
Growth media (minigut C)	50% (v/v) minigut A, 30% (v/v) minigut B, 20% (v/v) Rspondin1 enriched media, 1221.2 µg/mL nicotinamide, 163.19 µg/mL N-Acetyl-L-cysteine, 0.1 µg/mL human Noggin, 0.211 µg/mL A-83-01, 3.314 µg/mL SB202190, 0.05 µg/mL Human EGF and 0.021 µg/mL [Leu]15-Gastrin 1. Prepare fresh and sterile filter with 0.22 µm filter. Pre-warm at 37 °C for 15 minutes before use.

*Only needed if doing crypt isolation from frozen colonic biopsies.

### Protocol 4 Passaging colonoids

All equipment, materials, reagents, and prepared buffers needed for these procedures are listed in **Table 4**.

1. Always sterilize tools and plasticware with 70% EtOH before moving it into the hood. Place a 24-well plate in the incubator. Sterilize the laminar flow hood with 70% EtOH. Thaw Matrigel by placing the tubes on ice for 3-4 hours.
2. Once colonoids are ready for passaging, take the 24-well culture plate to a sterile laminar flow hood. Aspirate and discard the old media from all the wells without disturbing the Matrigel containing colonoids.
3. Place the 24-well plate on ice in an icebox.
4. Using a P1000 pipette, add 750 µL of minigut B containing 5% FCS with pressure on top of the Matrigel containing the colonoids to all the wells to break the Matrigel apart. Triturate minigut B containing 5% FCS and colonoids 5-6 times per well to dissolve the Matrigel. Collect the colonoids along with minigut B containing 5% FCS from all the wells into a 50 mL centrifuge tube that is placed on ice (*see* notes 14 and 15).
5. Add 750 µL of fresh minigut B containing 5% FCS to all the wells to wash and remove the leftover colonoids by scraping gently with a P1000 pipette tip (*see* notes 15-16). Pool this fraction of colonoids with colonoids collected in the previous step.
6. Spin the colonoids at 200 x *g* for 5 minutes at 4°C (*see* note 17).
7. Discard the supernatant completely and using a 10 mL pipette resuspend the pellet in 15 mL of TrypLE Express Enzyme (1x), phenol red containing 3.203 µg/mL Y-27632 (*see* note 18), pre-warmed at 37°C.
8. Incubate at 37 °C for 10 minutes. Homogenize and dissociate colonoids into single cells by carefully resuspending the colonoids with a sterile 20 mL syringe with blunt-ended 18-gauge needle up and down ten times (*see* notes 18-19).
9. Spin the single cells at 500 x *g* for 5 minutes at 4°C.
10. Discard the supernatant and resuspend the cells in 1 mL ice-cold minigut B with FCS.
11. Using Trypan blue stain, count the number of living cells/mL in a cell counter.
12. For freezing and biobanking, we aliquot 500 000 to 1 million cells in 1 ml FCS+5% DMSO and place the tubes in isopropanol boxes for 24 hours before we transfer the cells to liquid nitrogen.
13. For regular maintenance, colonoids are usually passaged every 7-10 days. We recommend to plate 15,000 cells in 50 µL of ice-cold Matrigel per well in the pre-warmed 24-well plates, for regular maintenance. For conducting experiments, resuspend 5,000-10,000 cells per 50 µL ice-cold Matrigel (*see* note 20) in each well of pre-warmed 24-well plates.
14. Incubate the plate containing single cells in Matrigel in the incubator for 20 minutes. Add 500 µL of minigut E on day 1 and day 3 of culture to the side of all the wells carefully without disturbing the Matrigel with cells. Further, refresh with minigut C every 2 days until the colonoids are ready for further passaging or differentiation to perform experiments.

**Table 4:** Passaging colonoids.

Equipment			
Water bath at 37 °C		Sterile 24-well flat bottom cell culture plates with lid	
Incubator at 37 °C with 5% CO_2_		Pipette, 10 mL	
Centrifuge at 4 °C for 50 mL tubes		Sterile centrifuge tubes, 50 mL	
Cell counter		Pipettes and filter tips, P200 and P1000	
Freezer -20 °C		Sterile plastic syringe, 20 mL	
		Ice and icebox	
		Microscope slide	
Materials and reagents			
Name		Catalog number	Manufacturer
Trypan blue			
Aliquots (1 mL) of Matrigel		CLS354230-1EA	Merck
Blunt fill needles 18-gauge		303129	BD Biosciences
Y-27632 - Prepare 3.2 mg/mL stock in DPBS, freeze 50 µL aliquots at -20 °C until use. See Note 18.		1254	Bio-Techne
TrypLE™ Express Enzyme (1x), phenol red		12605010	Thermo Fisher Scientific
Prepared buffers			
Name	Preparation		
Ice-cold minigut B with 5% FCS	Defrost aliquots from -20 °C (*see* notes 14 and 16). Add FCS to a final concentration of 5% v/v.		
TrypLE with ROCK-inhibitor	TrypLE™ Express Enzyme (1x), phenol red containing 3.203 µg/mL of the selective ROCK-inhibitor Y-27632.		
Growth media (minigut C)	50% (v/v) minigut A, 30% (v/v) minigut B, 20% (v/v) Rspondin1 enriched media, 1221.2 µg/mL nicotinamide, 163.19 µg/mL N-Acetyl-L-cysteine, 0.1 µg/mL human Noggin, 0.211 µg/mL A-83-01, 3.314 µg/mL SB202190, 0.05 µg/mL Human EGF and 0.021 µg/mL [Leu]15-Gastrin 1. Prepare fresh and sterile filter with 0.22 µm filter. Pre-warm at 37 °C for 15 minutes before use.		
Minigut E	Minigut C with 3.203 µg/mL of Y-27632. Freshly prepared, filtered with 0.22 µm filter. Pre-warm at 37 °C for 15 minutes before use.		
Freezing medium	FCS+5% DMSO. See Protocol 4, step 12.		

### Protocol 5 Differentiation of colonoids and treatment with IBD ligands; collection of conditioned media and processing colonoids for downstream assays

**Figure 2:**
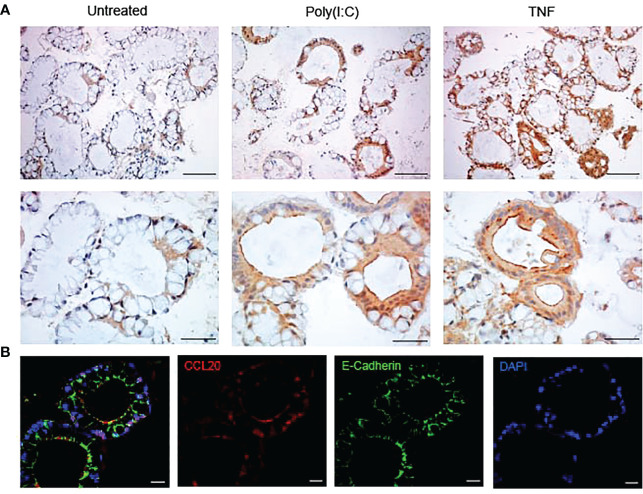
Colonoids stained with CCL20 by immunohistochemistry and immunofluorescence **(A)** Immunohistochemistry images of the expression of CCL20 protein in unstimulated colonoids and colonoids stimulated with Poly(I:C) and TNF for 24 hours. Upper panels are taken with 20x objective and the scale bars show 100 µm. Lower panels are taken with 40x objective and the scale bars show 50 µm. Images are obtained with Nikon Eclipse Ci microscope. **(B)** Immunofluorescence images of colonoids after 24-hour treatment with TNF showing CCL20 (in red), E-cadherin (in green) and DAPI (in blue). Images are taken at 63x objective with Zeiss 880 Airyscan confocal microscope and scale bars represent 20 µm.

**Figure 3:**
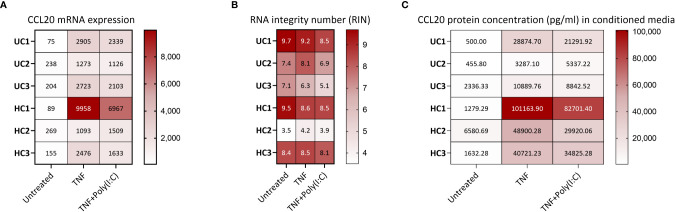
*CCL20* gene and protein expression from human colonoids **(A)** *CCL20* mRNA expression in colonoids from six donors (3 healthy controls (HC) and 3 ulcerative colitis (UC)) in untreated condition and after treatment with TNF or TNF+Poly(I:C) for 24 hours. Data are represented as normalized, log2 transformed mRNA reads obtained by gene expression analysis (bulk RNAseq, GEO accession GSE172404). The sequencing (75 cycles single end reads) was performed on an Illumina HiSeq4000 instrument, in accordance with the manufacturer’s instructions (Illumina), as described (20). LIMMA linear models identified differential gene expression between conditions with least square regression and empirical Bayes moderated t statistics. Correction-adjusted P-values ≤ 0.05 with Benjamin-Hochberg’s false discovery rate were statistically significant: TNF *vs.* Untreated (log_2_ Fold change 3.934, P=1.35E-15), TNF+Poly(I:C) *vs.* Untreated (log_2_ Fold change 3.621, P=1.45E-13). **(B)** For RNA-Seq of colonoids, the total RNA concentration was measured using a Qubit RNA HS Assay Kit on a Qubit 2.0 Fluorometer (Thermo Fisher Scientific, Waltham, MA, United States). Integrity was assessed using an Agilent RNA 6000 Nano Kit on a 2100 Bioanalyzer instrument (Agilent Technologies, Santa Clara, CA, United States). The heat map visualizes the RNA integrity number (RIN) for the different samples shown in 3A. **(C)** CCL20 protein release (pg/mL) from colonoids derived from the same six donors as in 3A-B. Conditioned media were collected from unstimulated colonoids, and colonoids stimulated with TNFand Poly(I:C) for 24 hours before CCL20 in conditioned media was measured using ELISA (**Supplementary protocol SP1**). The heat map shows individual concentrations. Both TNF and TNF+Poly(I:C) induced significant (*P*<0.05) enhanced release of CCL20 compared to untreated controls. Statistical analysis was performed on log_2_ transformed data using one way ANOVA followed by Dunnett’s multiple comparison test.

**Figure 4:**
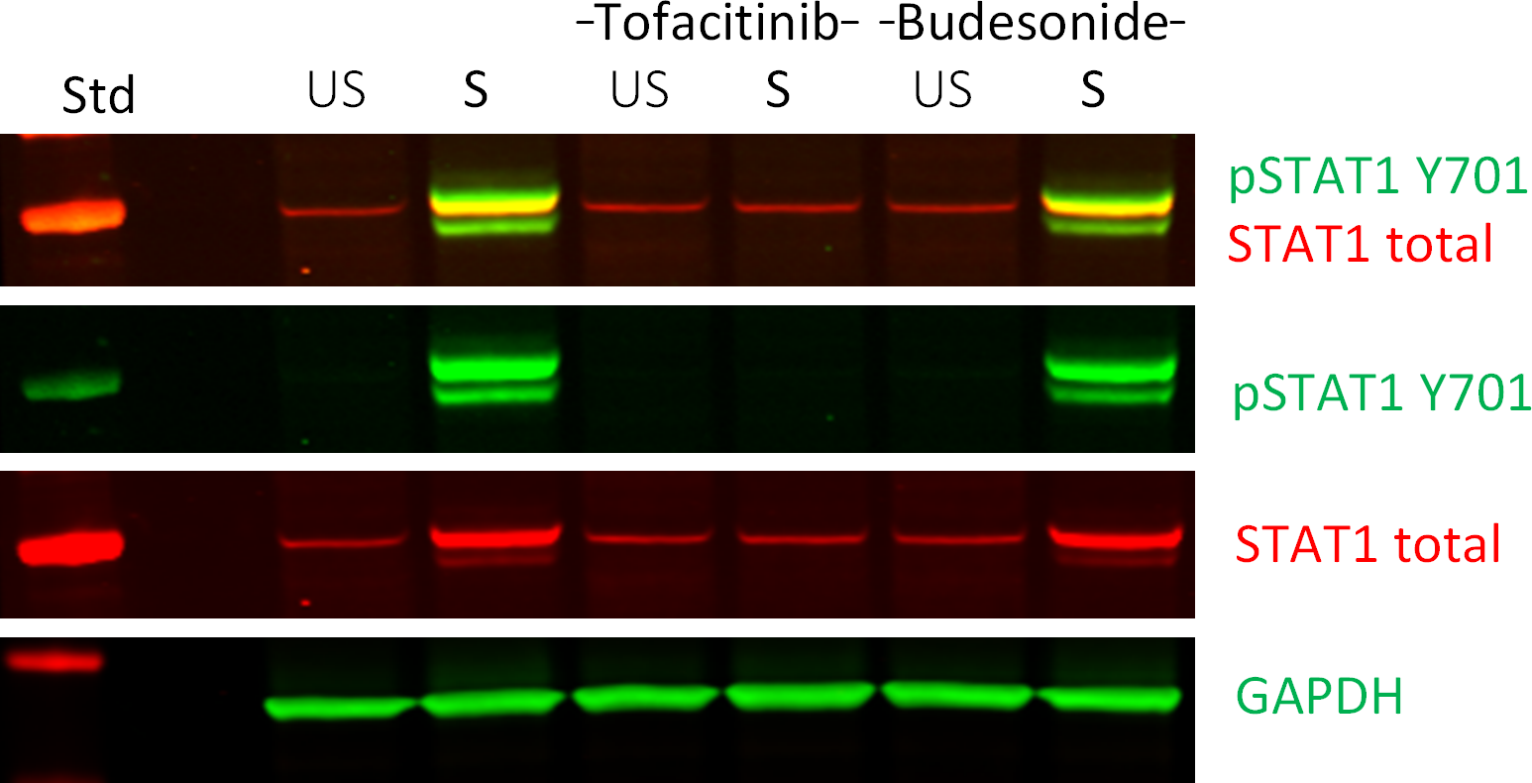
Western blot analysis of down-stream signaling in ligand and IBD-drug treated colonoids. Effect of Tofacitinib and Budesonide pre-treatment on TNF+Poly(I:C) stimulation in colonoids by immunoblotting, as described in (24) The IBD drug pre-treated colonoids were left unstimulated (US) or stimulated (S) with TNF+Poly(I:C). A merged image in the first row reveals overlap of signals (seen as orange) between phosphorylated (green) and total STAT1 (red) protein levels. The two next rows show the individual images from the merged image. GAPDH was used as a loading control. The left column shows protein standard (Std). The immunoblot is previously published as donor 3 in (24). The example image show proof of concept on how to use fluorescent secondary antibodies to visualize in a convincing way that the protein band signal of the phosphorylated STAT1 protein overlaps with the total STAT1 protein band signal, indicating that we are observing the correct immunosignal. The method is used in previous publications (25, 26).

The protocols describe a workflow (**Figure 1**) for differentiation of colonoids, treatment with e.g., IBD drugs and/or cytokines and processing for downstream assays. As proof of principle, we show data from 24 hours stimulation with TNF+Poly(I:C) with and without pre-treatments with IBD relevant drugs Tofacitinib and Budesonide (**Figures 2** **-** **4**). The colonoids are collected for immunostaining (Protocols 6 and 7, **Tables 6**, **7**; **Figure 2**), gene expression analysis (**Figure 3**), and Western blot (Protocols 8 and 9 and **Table 8**; **Figure 4**). Conditioned media is collected for examining e.g., chemokines secreted into the media by ELISA (Protocol 10, **Supplementary protocol SP1**; **Figure 3**). We usually collect the media and cells from 6 wells if we seed 10,000 cells per 50 µL Matrigel per well or from 12 wells if we plate 5000 cells per 50 µL Matrigel per well, for each ligand treatment (*see* note 21). All equipment, materials, reagents, and prepared buffers needed for these procedures are listed in **Table 5**.

1. Always sterilize tools and plasticware with 70% EtOH before moving it into the hood. After cells are passaged and seeded (Protocols 4), monitor seeded cells in growth media under a microscope every 2 days. On day 10, aspirate the growth media and add 500 µL pre-warmed differentiation media per well towards the side of the well of the plates (*see* note 20 and **Figure 1**).
2. On day 11, refresh the plates with 500 µL pre-warmed differentiation media per well and on day 14 refresh the plates with freshly prepared differentiation media without A-83-01 (*see* note 22). Preserve a certain volume of differentiation media for preparing the ligands on day 15.
3. On day 15, prepare the ligands and vehicle control (sterile water) in differentiation media. Working concentration of TNF is 0.1 µg/mL, and Poly(I:C) is 20 µg/mL, prepared in differentiation media. Add ligands and vehicle control to the plates such that the final volume of media with ligands is 500 µL per well. Dissolve the ligands evenly into the differentiation media by gently swirling the plate. Place the plate in the incubator. If doing drug experiments, Tofacitinib and/or Budesonide at desired final concentrations can be included in each of the refreshing differentiation media on days 11 and 14, and on day 15 one-hour pre ligand treatment, as previously described (24).
4. Twenty-four hours post-treatment with ligands, collect the conditioned media from all the wells into a 15 mL tube and place the tubes on ice (*see* note 21).
5. Place the empty plate immediately on ice once the supernatant is removed and add 500 µL of ice-cold cell recovery solution to all the wells.
6. Meanwhile, spin the conditioned media from colonoids at 800 x *g* for 10 minutes at 4°C to eliminate cell debris.
7. Make multiple aliquots of the supernatant containing conditioned media into sterile 1.5 mL tubes without disturbing the pellet (*see* note 23). Freeze the aliquots in -80°C until further use.
8. For collecting the colonoids present in cell recovery solution, using a P1000 pipette gently resuspend the colonoids 4-5 times with the cell recovery while trying to break the Matrigel apart. Collect the colonoids with the cell recovery solution into a 50 mL tube.
9. Refresh all the wells in the plate with 500 µL of ice-cold cell recovery solution, gently scrape the wells, collect the leftover colonoids from the plate, and pool them with the colonoids collected in the previous step.
10. Add 0.5 x volume of fresh ice-cold cell recovery solution to the volume that is present in the 50 mL tube containing the colonoids and mix well.
11. To provide good mixture of colonoids and cell recovery solution, incubate the colonoids on the ice at 45° angle for one hour with constant gentle shaking on an orbital shaker at 200-300 rpm.
12. Spin down the colonoids at 500 x *g* for 5 minutes at 4°C (*see* note 24).
13. If processing colonoids for immunostaining or RNA isolation:
14. Aspirate the supernatant without disturbing the pellet and add 10 mL ice-cold DPBS containing 0.1% BSA with full speed of the pipette directly on the pellet such that the pellet is released from the bottom of the tube. Adding DPBS containing 0.1% BSA with force should be sufficient to disperse and wash the pellet in the solution. Avoid trituration with the pipette several times.
15. Spin down the colonoids at 500 x *g* for 5 minutes at 4°C.
16. Aspirate the supernatant. Pre-wet P1000 tips with ice-cold DPBS containing 0.1% BSA and resuspend colonoid pellets in 1000 µL ice-cold DPBS containing 0.1% BSA and collect the colonoids into 1.5 mL microfuge tubes. Avoid trituration with the pipette several times.
17. Spin down the colonoids at 500 x *g* for 5 minutes at 4°C. Remove and discard the supernatant (*see* note 25).
  - Snap freeze the pellet using liquid nitrogen and freeze pellets immediately at -80°C until proceeding for mRNA isolation.
  - Alternatively, if processing pellets for immunostaining, proceed with Protocol 6.
18. If processing colonoids for Western blot analysis:
19. Aspirate the supernatant without disturbing the pellet and add 1 mL ice-cold cell recovery solution (*see* note 26).
20. Resuspend the pellet before transferring all the content to a pre-marked 1,5 mL Eppendorf tube (*see* note 27).
21. Spin down the colonoids at 500 x g for 5 minutes at 4°C.
22. Aspirate the supernatant without disturbing the pellet and add 1 mL ice-cold PBS with full speed of the pipette directly on the pellet such that the pellet is released from the bottom of the tube. Adding PBS with force should be sufficient to disperse and wash the pellet in the solution. Avoid touching the pellet with the pipette tip as the colonoids will stick to the plastic tip and be lost.
23. Spin down the colonoids at 500 x g for 5 minutes at 4°C. Remove and discard the supernatant.
24. Repeat pkt 22-23 a total of 3 times
25. Aspirate the supernatant and spin down once more without adding additional PBS. Remove all remaining PBS. Snap freeze the pellet using liquid nitrogen and freeze pellets immediately at -80°C until performing western blot protein analysis. *See* Protocol 9.

**Table 5:** Differentiation and treatment of colonoids and processing for downstream assays.

Equipment			
Orbital shaker		Sterile 24-well flat bottom cell culture plates with lid	
Incubator at 37 °C with 5% CO_2_		Pipette, 10 mL	
Centrifuge at 4 °C for 15 mL and 50 mL tubes		Centrifuge tubes, 15 mL and 50 mL	
Freezers -20 °C and -80 °C		Cryotubes, 1.5 mL	
		Sterile microfuge tubes, 1.5 mL	
		Pipettes and filter tips, P10, P200 and P1000	
		0.22 µm sterile filter	
		Ice and icebox	
Materials and reagents			
Name		Catalog number	Manufacturer
Recombinant human TNFα - Prepare 100 µg/mL stock in sterile water, freeze 50 µL aliquots at -20 °C until use.		300-01A	Peprotech
Poly(I:C) high molecular weight (HMW) - Prepare 1000 µg/mL stock in sterile water, freeze 500 µL aliquots at -20 °C until use.		tlrl-pic	*In vivo*gen
DAPT - Prepare 43.25 mg/mL stock in DMSO, freeze 10 µL aliquots at -20 °C until use.		2634	Bio-Techne
Cell recovery solution		734-0107	Corning
Tofacitinib - Prepare 150 mg/mL stock in DMSO, freeze 10 µL aliquots at -80 °C until use.		S5001	Selleck Chemicals
Budesonide - Prepare 150 mg/mL stock in DMSO, freeze 10 µL aliquots at -80 °C until use.		Batch S128602	Selleck Chemicals
DPBS, sterile and ice-cold		D8537-500ML	Merck
*Protein LoBind Eppendorf tubes, 1.5 mL		022431081	Eppendorf
Prepared buffers			
Name	Preparation		
DPBS with BSA	Sterile and ice-cold DPBS with 0.1% BSA.		
Differentiation media	5% (v/v) minigut A, 75% (v/v) minigut B, 20% (v/v) Rspondin1 enriched media, 163.19 µg/mL N-Acetyl-L-cysteine, 0.1 µg/mL human Noggin, 0.211 µg/mL A-83-01, 0.05 µg/mL Human EGF, 0.021 µg/mL [Leu]15- Gastrin 1 and 4.3 µg/mL DAPT. Prepare fresh and sterile filter with 0.22 µm cell filters. Pre-warmed for 20 minutes at 37 °C.		

*Only needed if processing for downstream Western blot.

### Protocol 6 Processing colonoids for immunostaining

Follow Protocol 5, step 1-17 and continue with the following procedure if collecting colonoids for immunostaining of paraffin sections. Embedding colonoid pellets in paraffin enables long term storage and multiple visualization analyses of the same colonoids from a specific experiment. Protocols for immunostaining and confocal imaging of intact 3D intestinal organoids are well described by others (27). All equipment, materials, reagents, and prepared buffers needed for the present procedure are listed in **Table 6**.

1. While samples are spinning in the centrifuge in step 17 of Protocol 5, thaw the HistoGel at 80°C in a thermomixer (*see* note 28).
2. Remove and discard the supernatant (*see* note 25) and place the tube with the pellet on ice.
3. Wear protective goggles before handling liquid nitrogen. Add 50-70 µL of the liquid HistoGel using a P200 pipette to the pellet. Using another P200 Pipette with a volume of 120-150 µL and cut tips, triturate once or twice without creating bubbles, take the entire HistoGel with the colonoids, and pipette it onto a microscopic slide pre-cooled in liquid nitrogen (*see* note 29).
4. Place the slide on top of the liquid nitrogen for about 1-2 minutes to allow for the solidification of the HistoGel containing the colonoids.
5. Slide the frozen HistoGel into a paraffin embedding cassette using forceps, close the cassette and place the cassette in the formalin fixative (*see* note 30).
6. After overnight-24 hours of fixation at room temperature, embedding cassettes with the samples are dehydrated and cleared with the following protocol in a histology processing machine.
  - Wash once in 70% EtOH for 20 min.
  - Wash once in 80% EtOH for 20 min.
  - Wash twice in 96% EtOH for 20 min each.
  - Wash twice in 100% EtOH for 20 min each.
  - Wash twice in Neo-Clear for 20 min each.
  - Wash three times in paraffin for 1 hour each.
7. Transfer the samples to a tissue embedding machine and prepare standard paraffin blocks. Preferably store in the dark at room temperature until use.

**Table 6:** Processing colonoids for immunostaining.

Equipment		
Curved steel forceps	Paraffin-embedding cassettes	
Long steel tweezers	Pipettes and tips, P200 and P1000	
Two pair of protective goggles	Cut P200 tips	
Thermomixer at 80 °C	Liquid nitrogen	
Histology processing machine		
Tissue embedding machine		
Materials and reagents		
Name	Catalog number	Manufacturer
EtOH (Absolute, 95, 80 and 70%)		
Paraffin		
Superfrost plus microscope slides	10149870	Fisher Scientific
Neo-Clear Xylene Substitute	1098435000	Merck
Formaldehyde 4% stabilized, buffered (pH 7,0 ± 0,1)	9713.1000	VWR
Thermo Scientific Richard-Allan Scientific HistoGel Specimen Processing Gel	HG-4000-012	Fisher Scientific (*see* note 28)

### Protocol 7 Immunohistochemical and immunofluorescent staining of colonoids

These protocols have been optimized for staining of paraffin sections using the specific CCL20 antibody. The principle is similar for any other antibody, but with optimalisation of antigen retrieval, antibody dilution and incubation. All equipment, materials, reagents, and prepared buffers needed for these procedures are listed in **Table 7**.

1. Cut out paraffin sections of 4 µm thickness with a microtome and float them on a water bath with distilled water maintained at 40°C.
2. Carefully gather the sections onto a superfrost plus microscope slide by inserting the slide underneath the section and lifting them off the water bath. Drip off excess water from the slides.
3. Dry slides overnight at room temperature and store them in light proof boxes preferably at 4°C until used for staining.
4. On the day of the staining procedure, place the slides in a microscope slide staining rack and incubate at 60°C for 30-60 minutes to melt the paraffin. Let the tissues cool down to room temperature for about 5 minutes (optional) and proceed with deparaffinization.
5. Prepare and keep ready slide staining jars with solutions needed to deparaffinize, rinse, block endogenous peroxidase activity, washing with buffer and nuclear counterstaining with Hematoxylin inside a fume hood.
6. To deparaffinize, put the slides in Neo-Clear, agitate by lifting the slide rack up and down in the jar a few times and incubate for 10 min (*see* note 31). Repeat this step once more with fresh Neo-Clear.
7. Rinse in a series of decreasing EtOH concentrations in the following order: absolute EtOH, 95% EtOH, 80% EtOH and 70% EtOH for one minute with occasional agitation in each EtOH concentration.
8. If performing immunohistochemistry:
9. To block endogenous peroxidase activity, rinse in distilled water for one minute before incubating the slides for 10 minutes in 3% hydrogen peroxidase solution kept in the dark. Rinse in distilled water for one minute (*see* note 32).
10. Transfer the slides to a microwave-compatible staining rack and jar. Perform antigen retrieval by boiling the slides at approximately 90-160 W for 15 minutes in the Tris EDTA buffer pH 9 in a microwave oven. Allow the slides to cool down to room temperature in the same buffer for about 20-30 minutes (*see* note 33).
11. Place the slides back in slide staining rack and rinse twice in wash buffer for 5 minutes each wash. Optional: incubate slides with blocking buffer for 30 minutes at room temperature followed by washing once with wash buffer for 5 minutes.
12. Place slides horizontally. Prepare enough volume of CCL20 antibody in antibody diluent (1:300) for all slides. Add enough diluted antibody to completely cover the colonoid pellet in each section (*see* note 34), carefully add the GelBond film and incubate slides overnight at 4°C in a humid chamber. For all incubations done in a humid chamber: Use lint-free tissue paper to dry off excess wash buffer on the slide around the colonoid pellet and place the slides in the chamber. Do not touch the colonoid pellet and make sure it does not dry out (*see* note 35).
13. The following day: place the slides back in a slide staining rack and wash two times in wash buffer for 5 minutes each time with occasional agitation. The GelBond film will slide off when the slides are agitated in wash buffer.
14. Place slides horizontally. Add enough volume of the secondary antibody rabbit/mouse EnVision-HRP/DAB+ reagent to completely cover each section and incubate in a humid chamber for 30 minutes at room temperature.
15. Wash slides two times in wash buffer for 5 minutes each time with occasional agitation. Place slides horizontally.
16. Add the Dako Liquid DAB+ Substrate Chromogen to completely cover each section. Prepare a master mix of the required amount by mixing 1:50 of DAB+ Chromogen and the substrate buffer.
17. Incubate for approximately 1 minute at room temperature. DAB incubation time will vary depending on antigen and antibody. View under the microscope and stop the reaction by placing the slide in distilled water. Further, wash the slides in water for 5 minutes.
18. Prewarm the mounting media by placing at 60°C for 5-10 minutes.
19. Perform nuclear staining by incubating slides in Hematoxylin stain for 10 seconds.
20. Rinse the slides for 2 minutes in a container under running tap water (not directly on the slides) at lukewarm temperature. Dry the slides with paper towels.
21. To mount the slides, place slides horizontally, and add one drop of the mounting media without creating bubbles and carefully place the coverslip on top of the sections and gently press with an applicator stick (*see* note 36). Representative images of the expression and localization of CCL20 in colonoids unstimulated and stimulated with Poly(I:C) and TNF are shown in **Figure 2A**.
22. If performing double immunofluorescence:
23. Follow the manufacturer’s instructions for the MaxDouble M488&R650 ImmunoFluorescence Double Staining Kit for human tissue with minor modifications as described below. Here, we describe the protocol optimized for the rabbit CCL20 antibody in combination with the mouse E-Cadherin antibody described in **Table 7**.
24. Use lint-free tissue paper to dry off excess liquid around the colonoid pellet and place the slide horizontally. To block autofluorescence, add enough MaxBlock Autofluorescence Reducing Reagent (Reagent 1) to completely cover the pellet (*see* note 34) and incubate for 5 minutes in room temperature (*see* note 37).
25. Place the slides back in slide staining rack and rinse in 60% EtOH for 1 minute with occasional agitation, followed by rinsing in distilled water for 5 minutes and wash buffer for 5 minutes.
26. Perform antigen retrieval as per step 10.
27. Place the slides back in a slide staining rack and rinse twice in wash buffer for 5 minutes each wash. Optional: Incubate slides with blocking buffer for 30 minutes at room temperature followed by washing once with wash buffer for 5 minutes.
28. Place slides horizontally. Prepare sufficient volume of mixed CCL20 antibody (1:300 dilution) and E-cadherin antibody (1:600 dilution) in antibody diluent. Add 70-100 µL diluted antibody mix to completely cover each section, carefully add GelBond film and incubate slides overnight at 4°C in a humid chamber (*see* notes 34-35).
29. The following day: place the slides back in slide staining rack and wash two times in wash buffer for 5 minutes each time with occasional agitation. The GelBond film will slide off when the slides are agitated in wash buffer.
30. Place slides horizontally. Mix equal amounts of Mouse Signal Amplifier (Reagent 2A) and Rabbit Signal Amplifier (Reagent 2B) (*see* note 38), add enough volume to completely cover each section and incubate in a humid chamber for 30 minutes at room temperature.
31. Wash slides two times in wash buffer for 5 minutes each time with occasional agitation.
32. Place slides horizontally. Mix equal amounts of MaxFluor 488 labelled linker for mouse (Reagent 3A) and MaxFluor 650 labelled linker for rabbit (Reagent 3B) (*see* note 38), add enough volume to completely cover each section and incubate in a humid chamber for 60 minutes at room temperature kept in the dark (*see* note 38).
33. Wash slides two times in wash buffer for 5 minutes each time with occasional agitation kept in the dark.
34. Place the slides horizontally, add enough Post-Detection Conditioner (Reagent 4) to completely cover each section and incubate for 5 minutes at room temperature kept in the dark.
35. Wash slides two times in distilled water for 5 minutes each time with occasional agitation kept in the dark.
36. Prepare enough volume of DAPI in antibody diluent (1:1000) for all slides. Place the slides horizontally and perform nuclear staining by adding enough volume to completely cover each section and incubate for 5 minutes at room temperature kept in the dark (*see* note 40).
37. Mount the slides as step 21. Wrap in aluminum foil and store in the dark at 4°C. Equilibrate to room temperature 30-60 min before visualizing under a fluorescent or confocal microscope with proper filter settings (*see* note 41). Representative images of the expression and localization of CCL20 (red) and E-cadherin (green) alone or co-localized (yellow), in colonoids stimulated with TNF for 24 hours are shown in **Figure 2B**.

**Table 7:** Immunohistochemistry and immunofluorescence of paraffin sections of colonoids.

Equipment			
Microtome		Microscope slide staining racks and jars, at least one set needs to be microwave-compatible	
Histology water bath at 40 °C		Microfuge tubes, 1.5 mL	
Fume hood		Pipettes and tips, P10, P200, and P1000	
Humid chamber			
Fridge or cold room at 4 °C			
Incubator at 60 °C			
*Light microscope, Nikon Eclipse Ci			
^§^Confocal microscope, Zeiss 880 Airyscan Confocal Microscope			
Materials and reagents			
Name		Catalog number	Manufacturer
Superfrost plus microscope slides		10149870	Fisher Scientific
Neo-Clear Xylene Substitute		1098435000	Merck
Ethanol (Absolute, 95, 80 and 70%)			
Distilled water			
*Hydrogen peroxide 30%		1072091000	Merck
^§^4′,6-diamidino-2-phenylindole (DAPI) (1 mg/mL). Dilute 1:1000 in antibody diluent before use.		62248	Thermo Fisher Scientific
^§^MaxDouble M488&R650 ImmunoFluorescence Double Staining Kit for human tissue		DSMR-H3	MaxVision Biosciences Inc
*Dako REAL EnVision Detection System, Peroxidase/DAB, Rabbit/Mouse, HRP		K500711-2 (RRID : AB_2888627)	Agilent
Anti CCL20 (rabbit polyclonal antibody). Dilute 1:300 in antibody diluent before use		ab9829 (RRID: AB_308798)	Abcam
Anti E-Cadherin (mouse monoclonal antibody). Dilute 1:1000 in antibody diluent before use.		610182 (RRID: AB_397581)	BD Biosciences
*Glycergel Mounting Medium, Aqueous		C0563	Agilent
GelBond film		53746	Lonza
Cover Glass		631-0158	VWR
Applicator stick		120760	Selefa
Prepared buffers			
Name	Preparation		
*Hydrogen peroxide 3%	Dilute 30% hydrogen peroxide 1:10 with distilled water. Cover with aluminum foil to keep it in the dark.		
Tris EDTA pH 9 buffer	1.21 g of Tris Base, 0.37 g of EDTA prepared in 1000 mL distilled water, adjusted to pH 9.		
10X and 1x TBS buffer	9.7 g Tris-Base, 66.12 g Tris-HCL, 87.68 g NaCl prepared in 1000 mL distilled water, adjusted to pH 7.4. Store at 4 °C and dilute with distilled water to prepare 1x TBS.		
Wash buffer	1x TBS with 0.05% (v/v) Tween-20 (TBST).		
Blocking buffer	1x TBS with 3% (w/v) BSA.		
Antibody diluent	1x TBS with 0.025% (v/v) Tween-20 and 1% (w/v) BSA.		
*Hematoxylin stain	Mix 2.5 g of hematoxylin dye with 100 mL glycerol, 50 mL glacial acetic acid, and 200 mL sterile water. Add 0.25 g of periodic acid to this mixture and set aside. In another beaker, mix 25 g powdered aluminum sulphate with 100 mL lukewarm sterile water. Mix the contents of the two beakers and filter with Whatman filter paper and store in a dark bottle.		

*Only needed if doing immunohistochemistry. ^§^ Only needed if doing immunofluorescence.

### Protocol 8 RNA isolation from colonoids

Colonoid pellets are prepared as per Protocol 5 and frozen in -80 °C. To isolate RNA from colonoids, we use the RNeasy Mini kit from Qiagen with minor adjustments to the protocol as described below. All equipment, materials, reagents, and prepared buffers needed for these procedures are listed in **Table 8**.

1. The entire procedure is done inside a laminar flow hood. Sterilize and prepare the laminar flow hood for the procedure.
2. Prepare 1 mL syringes with 20-gauge needles and leave them aside until use.
3. Calculate and prepare the amount of lysis buffer required depending on the number of samples. We recommend using 600 µL of lysis buffer (containing 1% BME) per colonoid pellet collected (*see* note 42).
4. Thaw the frozen colonoid pellets on ice.
5. Add 600 µL of lysis buffer containing BME and triturate the samples with P1000 pipette for 3-4 times and move to the next sample and perform the same to all the samples (*see* note 43).
6. Homogenize the lysates by passing through 1 mL sterile syringe (containing 20-gauge needles) ten times to get a homogenous lysate.
7. Take 300 µL of the lysate and mix with 300 µL of 70% EtOH, mix thoroughly (*see* note 44) and add them to RNeasy spin column placed on top of a 2 mL collection tube. Repeat for all the samples.
8. Close the lid of RNeasy spin columns, spin at 10,000 x *g* for 30 seconds.
9. Discard the flow-through (*see* note 45). Take rest of the homogenized colonoid lysate (approximately 300 µL), mix thoroughly with 300 µl of 70% EtOH and quickly transfer to RNeasy spin column used in step 7. Repeat for all the samples.
10. Close the lid of RNeasy spin column, spin at 10,000 x *g* for 30 seconds. Discard the flow-through.
11. Add 700 µL of buffer RW1 to all the RNeasy spin column, spin at 10,000 x *g* for 30 seconds. Discard the flow-through and place the RNeasy spin column back into the collection tube.
12. Add 500 µL of RPE buffer to all the RNeasy spin column, spin at 10,000 x *g* for 30 seconds. Discard the flow-through and place the RNeasy spin column back into the collection tube.
13. Add 500 µL of RPE buffer to all the RNeasy spin column and spin at 10,000 x *g* for 2 minutes. Discard the flow-through with the collection tube and place the RNeasy spin column into a new 2 mL collection tube, while ensuring that there is no left-over buffer clinging outside the filter.
14. Spin at 10,000 x *g* for 2 minutes to remove any leftover buffer from previous washes and to dry the spin column. Discard the collection tube with any leftover flow-through.
15. Place the spin column on top of a new 1.5 mL microfuge collection tube with a lid, provided with the kit.
16. Add 30 µL of RNase-free water (provided with the kit) directly onto the spin column membrane (*see* note 46). Close the lid of the spin column and incubate at room temperature for 5 minutes.
17. Spin at 10,000 x g for one minute. Now the eluted RNA will be in the collection tube.
18. In order to increase the yield of the RNA, remove the eluate from the 1.5 mL microfuge tubes and run it through the spin column again by adding eluate directly to the membrane of the spin column placed on top of the 1.5 mL microfuge tube, incubate at room temperature for 5 minutes and spin at 10,000 x *g* for 1 minute.
19. Measure the concentration of the eluted RNA using a Nanodrop instrument.
20. On an average, for experiments performed in 24-wells plates, we get 10-20 µg of RNA yield out of colonoid pellets collected from 6 wells (initial plating density of 10,000 cells per well) or 12 wells (initial plating density of 5000 cells per well).
21. Store the RNA at -80°C, until used for gene expression analysis by e.g., real time polymerase chain reaction or gene sequencing (RNA-seq). An example of *CCL20* mRNA expression detected by RNA-seq is shown in **Figure 3A**. For RNA-Seq of colonoids, the total RNA concentration was measured using a Qubit RNA HS Assay Kit on a Qubit 2.0 Fluorometer (Thermo Fisher Scientific, Waltham, MA, United States). Integrity was assessed using an Agilent RNA 6000 Nano Kit on a 2100 Bioanalyzer instrument (Agilent Technologies, Santa Clara, CA, United States) (**Figure 3B**).

**Table 8:** Performing Western blot and RNA isolation on colonoids.

Equipment			
*Laminar flow hood		*Sterile syringes, 1 mL	
*Nanodrop (microvolume spectrophotometer)		Pipettes and filter tips, P10, P200 and P1000	
Minishaker (M2 Minishaker, IKA)		*Ice and icebox	
LI-COR Odyssey Imager and Image Studio Software			
Materials and reagents			
Name		Catalog number	Manufacturer
Frozen colonoid pellets (see Protocol 5)			
*EtOH, 70% freshly prepared with deionized water and absolute EtOH			
*20-gauge needles		305175	BD Biosciences
*RNeasy Mini kit		74106	Qiagen
*2-Mercaptoethanol (BME)		63689	Sigma-Aldrich
NaCl, 1 M			
Tris-HCL, 1M pH 7.5			
Dithiothreitol (DTT), 1M			
Methanol (MeOH)			
1x TBS buffer			
Ultrapure EDTA (0.5M)		15575-038	Invitrogen
Complete^®^ EDTA-free protease inhibitor		11873580001	Sigma-Aldrich
Phosphatase inhibitor cocktail I (PIC1)		P2850	Sigma Aldrich
Phosphatase inhibitor cocktail II (PIC2)		P0044	Sigma Aldrich
NP-40		492018	Sigma Aldrich
Pierce BCA protein assay kit		23225	Thermo Fisher Scientific
NuPage LDS Sample buffer (4X). Dilute to 1x before use		NP0007	Invitrogen
NuPage MOPS SDS running buffer (20X). Dilute to 1x before use		NP0001	Invitrogen
NuPage Transfer buffer (20X). Dilute to 1x before use		NP00061	Invitrogen
4-12% Nupage Bis-Tris gel		NP0321BOX	Invitrogen
Nitrocellulose membrane 0,2 µm		1620112	Bio-Rad
XCell SureLock Mini-Cell Electrophoresis System		EI0001	Thermo Fisher Scientific
XCell II Blot Module		EI9051	Thermo Fisher Scientific
Blocking Buffer for fluorescent Western blot		MB-070	Rockland
Anti Phospho-STAT1 (Tyr701) (rabbit monoclonal antibody). Dilute 1:1000 in TBST before use		7649S (RRID : AB_10950970)	Cell Signaling Technology
Anti STAT1 (mouse monoclonal antibody). Dilute 1:1000 in TBST before use		9176S (RRID : AB_2240087)	Cell Signaling Technology
Anti GAPDH XP (rabbit monoclonal antibody). Dilute 1:5000 in TBST before use		5174S (RRID : AB_10622025)	Cell Signaling Technology
Goat a-Rabbit IgG (H&L) Secondary ab. Dylight 800 4xPEG. Dilute 1:5000 in TBST before use		SA5-35571 (RRID : AB_2556775)	Thermo Fisher Scientific
Goat anti-mouse IgG (H&L) Secondary ab. Dylight 680 conjugated. Dilute 1:5000 in TBST before use		35518 (RRID : AB_614942)	Thermo Fisher Scientific
Prepared buffers			
Name	Preparation		
Buffer I	Mix with distilled water to a final concentration of 50 mM Tris-HCl, pH 7,5, 150 mM NaCl, 1 mM DTT, 1x Complete, 1x PIC1 and 1x PIC2		
Buffer II	Mix with dH2O to a final concentration of 50 mM Tris-HCl, pH 7,5 and 150 mM NaCl, 10 mM EDTA, 2% (v/v) NP-40, of 1 mM DTT, 1x Complete, 1x PIC1 and 1x PIC2		
Denaturation buffer	1x LDS sample buffer with 40 mM DTT		
TBST	1x TBS with 0.05% Tween-20		

*Only needed if doing RNA isolation.

### Protocol 9 Western blot protein analysis

For detection of intracellular signaling in colonoids, we recommend Western-blot analysis of protein isolated from harvested colonoids, as described in Protocol 5. The present protocol is optimized for detection of Phospho-STAT1 and STAT1 total by Western blot using RIPA lysis buffer (*see* note 47) and the specific Phospho-STAT1 and STAT1 total antibodies. All equipment, materials, reagents, and prepared buffers needed for these procedures are listed in **Table 8**. Example blot is shown in **Figure 4**.

1. Thaw cell pellets from Protocol 5 on ice.
2. Spin down the colonoids at 500 x *g* for 5 minutes at 4°C.
3. Resuspend in 1x pellet-cell-volume (PCV) ice-cold Buffer I until the pellet is completely dissolved.
4. Add 1xPCV ice-cold Buffer II and resuspend until the pellet is completely dissolved. Be careful to avoid air bubbles (*see* note 48).
5. Place the tubes on a shaker at 1100 rpm at 4°C for 2 hours.
6. Centrifuge at 13000 rpm at 4°C for 20 min. The supernatant is the protein extract.
7. Determine the protein concentration by the bicinchoninic acid protein assay (BCA) technique (28).
8. Denature 30 µg protein lysates in denaturation buffer for 10 minutes at 70°C before separation on 4–12% NuPage Bis-Tris gels and electroblotting onto nitrocellulose membranes.
9. The volume of protein lysate (in µl) from the BCA assay calculated to have 30 µg protein is mixed with denaturation buffer and added the required volume of 10 mM Tris HCl, 10 mM NaCl to a final volume of 24 µl. Denature the samples for 10 minutes at 70°C (*see* note 49).
10. The denatured samples are separated on 4–12% NuPage Bis-Tris gels in 1x MOPS running buffer at 180 V for 1.5 hours (*see* notes 50-51).
11. The proteins are electroblotted onto nitrocellulose membranes in Transfer buffer with 10% (v/v) MeOH at 40 V for 1 hours (*see* notes 50-51).
12. Let the membranes dry before continuing. This will fixate the proteins to the membrane (*see* note 52).
13. Block the membranes in Rockland Blocking Buffer for 1 hour at room temperature, protected from direct light.
14. Remove the blocking buffer (*see* note 53).
15. Incubate overnight at 4°C with the rabbit primary antibody Phospho-STAT1 diluted 1:1000 in TBST as suggested in the datasheet by the manufacturer.
16. Wash the membrane 3x 10 min with TBST
17. Incubated with Dylight 800 secondary anti-rabbit antibody (green fluorescence) diluted 1:5000 in TBST for 1 hour at room temperature protected from direct light.
18. Wash the membrane 2x 10 min with TBST and 1x 10 min with TBS (*see* note 54).
19. Images are obtained with LI‐COR Odyssey and analyzed using Image Studio Software (LI‐COR Biosciences, NE, USA).
20. Incubate overnight at 4°C with the mouse primary antibody STAT1 total diluted 1:1000 in TBST as suggested in the datasheet by the manufacturer.
21. Wash the membrane 3x 10 min with TBST
22. Incubated with 680 Dylight anti-mouse secondary antibody (red fluorescence) diluted 1:5000 in TBST for 1 hour at room temperature protected from direct light (*see* note 55-56).
23. Wash the membrane 2x 10 min with TBST and 1x 10 min with TBS (*see* note 54).
24. Images are obtained with LI‐COR Odyssey and analyzed using Image Studio Software (LI‐COR Biosciences, NE, USA).
25. Incubate overnight at 4°C with the rabbit primary antibody GAPDH (housekeeping protein) diluted 1:5000 in TBST as suggested in the datasheet by the manufacturer (*see* note 57).
26. Wash the membrane 3x 10 min with TBST
27. Incubated with Dylight 800 secondary anti-rabbit antibody (green fluorescence) diluted 1:5000 in TBST for 1 hour at room temperature protected from direct light.
28. Wash the membrane 2x 10 min with TBST and 1x 10 min with TBS (*see* note 54).
29. Images are obtained with LI‐COR Odyssey and analyzed using Image Studio Software (LI‐COR Biosciences, NE, USA).

### Protocol 10 ELISA to examine CCL20 release in conditioned media from colonoids

In principle, anything secreted from the cultured colonoids can be measured in the harvested conditioned media (Protocol 5). To measure cytokines/chemokines we often use solid-phase Sandwich ELISA kits from R&D Systems (Abington, United Kingdom), following the manufacturer’s instructions. An example protocol (including notes) for measurement of CCL20 release to the conditioned media upon treatment with IBD-relevant ligands such as TNF and Poly(I:C) is given in the **Supplementary protocol SP1**. Data from analysis using ELISA kit CCL20 (DY360) from R&D Systems are shown in **Figure 3C**.

## Notes and troubleshooting for the procedures given in Protocols 1-10

### Notes to Protocol 1: Wnt-3A enriched media (minigut A) and Rspondin1 enriched media

1. Centrifugation at a low speed of 125 x *g* may not pellet the cells entirely towards the bottom of the tube, it is therefore crucial to aspirate supernatant carefully without disturbing the cell pellet.
2. During trypsinization process, after incubation of flask at 37 °C for 5 minutes, tap the flask gently to aid with the dissociation of cells, and place the flask back into the incubator for 5 more minutes at 37 °C. Follow the trypsinization under a microscope every 5 minutes.
3. Working with multi-layer flasks reduces time and use of plastic, but it can be challenging to handle them. It is essential to equilibrate the flasks properly, so the cells in all the layers receive the same amount of media. Keep track of the flasks every day by checking media levels in the different layers of the flasks. When adding the media into the mixing port of the flask, keep the flask vertical. Turn the flask such that the front side of the flask is facing towards you. Tilt the flask right side to 45° angle and wait for a few seconds until the media is evenly spread across the five layers in the corner of the flask. Then, directly place the flask horizontally before moving it to the incubator. When removing media, invert the flask to release the contents.
4. If more conditioned media is not desired, either freeze down cells for preservation or stop plating cells in complete growth media in step 9 in Protocol 1 and step 3 in Protocol 2 (flasks A and B). To freeze cells, perform the following steps: wash with 10 mL DPBS, trypsinate with 2 mL trypsin-EDTA. After trypsinization, add 10 mL complete growth media to stop the trypsinization and spin cells at 400 x *g* for 5 minutes at 4°C. Aspirate the supernatant, count cells and resuspend pellet in FCS with 5% DMSO and transfer cryotubes with cells to isopropanol boxes to freeze cells at -80°C. After 24-48 hours, transfer them to liquid nitrogen for long-term preservation. It is vital to freeze cells from earlier passages as later passage of cells have occasionally shown improper growth of cells or loss of Wnt-3A activity. It is also advisable to culture cells from the same passage used for producing enriched media, in the media containing the selection antibiotics. Lack of cell growth in these flasks indicate loss of activity of Wnt-3A-encoding plasmid.
5. We have experienced that the 293-HA-Rspol-Fc cells may sometimes grow slower than what is described in the protocol. However, the cells must be fully confluent before passaging and before adding the AD-DF++ media. Full confluency will lead to media with optimal Rspondin1 concentration for colonoid growth.
6. We have also observed that the cells may float in the AD-DF++ media after 5-6 days. However, these cells are removed during the centrifugation process, and this does not impact the growth of colonoids.

### Notes to Protocol 3: Isolation of crypts from fresh and frozen colonic biopsies

7. Maintain DPBS, chelation buffer with and without EDTA, ice-cold at all times.
8. Since pinch biopsies are collected with standard colonoscopy forceps, the biopsy often has a C shape. When pinning the biopsies to the silicone coated petri dish, the convex side should be facing upwards, and the concave side of the biopsy should face downwards towards the petri dish.
9. When aspirating the chelation buffer containing EDTA from the petri dish, it is critical not to disturb the biopsies or be harsh when washing with the chelation buffer. While washing with the chelation buffer, do not directly add the buffer on top of the biopsies. Since the crypts are not tightly adhered to the biopsy after EDTA treatment, you may destroy or lose crypts. Therefore, wash the biopsies with the chelation buffer by adding the chelation buffer to a region of the petri dish where the biopsies are not pinned down.
10. After filtering, check flow-through for crypt fractions using a microscope.
11. After spinning the crypts fraction at 50 x *g*, the pellet may not settle down completely. It is critical not to discard crypts during this step. If unsure, you can take a small volume of the supernatant to check for the absence of crypts. If the centrifugation process is improper, the speed may be increased up to 200 x *g*.
12. While working with Matrigel, it is essential to be quick and careful not to introduce bubbles. Matrigel must be ice-cold at all times and long preparation time at room temperature can solidify the gel, making it inconvenient to work with it. Matrigel from Corning comes in 10 mL volumes. Place on ice overnight at 4°C to thaw the Matrigel. Swirl the Matrigel bottle carefully without creating bubbles and triturate several times carefully with a P1000 pipette without creating bubbles. Make 1 mL aliquots in sterile 1.5 mL microfuge tubes and store at -20°C until use.
13. Usually, we plate 200-500 crypts per 50 µL Matrigel. In our hands, our judgement is that high crypt density enhances the probabilities to successfully establish colonoids from biopsies. We recommend to look at the density of crypts in a microscope before seeding the cells.

### Notes to Protocol 4: Passaging colonoids

14. Minigut B with 5% FCS has to be ice-cold throughout the process to get the best disruption of Matrigel.
15. It is vital to place the 24-well plate on ice during this step to avoid re-solidification of Matrigel in the minigut B with FCS.
16. More than 90% of the colonoids come off the plate during the first round of collection with 750 µL of minigut B containing 5% FCS. Avoid resuspending more than 5-6 times as this could delay preparation time that may lead to reduced viability of the cells.
17. Since colonoids are spun at a very low speed, the pellet may not adhere to the bottom of the 50 mL tube. However, the pellet will clump together, and it is important not to dissociate or accidentally discard the pellet.
18. We recommend using 15 mL of TrypLE Express Enzyme (1x) containing 3.203 µg/mL Y-27632 (ROCK inhibitor) for dissociating colonoids collected from one 24-well plate. TrypLE Express enzyme can clump colonoids together. However, when dissociation is performed with the syringe and needle, the colonoids separate into single cells. Both Thiazovivin and Y-27632 are used to prevent anoikis in cultures with single cells and will probably work equally well for establishment and passaging of colonoids (29).
19. Add 10 µL of the dissociated cells on a microscope slide to check for dissociation efficiency. If required, more dissociation with the syringe and needle may be performed to obtain single cells. However, repeated dissociation with the syringe and needle can cause cell death.
20. Our experience is that the growth rate (i.e days to form colonoids) is the same when seeding different cell numbers, but there is a critical limit to how few cells we can seed. We have by trial-and-error landed on minimum 5000 and maximum 10 000 cells per 50 ul Matrigel for the 14-15 days experimental setup described. However, there are certainly donor differences, and some donors form larger colonoids faster than others. Such inter-individual differences in expansion ability or “stemness” is also observed by Pleguezuelos‐Manzano et al. (27). We have tested thawing several passages from various donors and patient-specific growth patterns such as colonoid morphology appear coherent when cultured from different passages. It is essential to make sure there is sufficient growth of the colonoids before starting differentiation. If growth is slow, maintain the colonoids in minigut C for another day or two. However, keeping the colonoids too long in culture can cause the Matrigel to disintegrate, and the colonoids to float in the media and die.

### Notes to Protocol 5: Differentiation of colonoids and treatment with IBD ligands; collection of conditioned media and processing colonoids for downstream assays

21. It is important to collect media and cells only from those wells where the colonoids have grown sufficiently throughout the Matrigel dome. Less number of colonoids can impact protein studies involving ELISA.
22. Addition of A-83-01 factor inhibits Transforming growth factor beta (TGF-β) signaling. If your experiments involve investigation of pathways related to TGF-β signaling, it is advisable to remove A-83-01 from the differentiation media on day 14.
23. Pellet is often not visible to the naked eye, so it is important not to disturb the bottom of the tube after centrifugation. Conditioned media collected in step 4 may be preserved on ice until the completion of step 10 in Protocol 5 to save time during the procedure.
24. To reduce processing time, equip the laminar flow hood with several 10 mL serological pipettes, particularly when processing multiple samples.
25. It is important to aspirate the supernatant completely. It is often easier to remove the last bit of the supernatant with a P200 pipette.
26. Fill and eject pipette tips with Cell Recovery Solution a couple of times before adding 1 mL to the cell pellet
27. It might be helpful to use low binding protein Eppendorf tubes (**Table 5**) to prevent the colonoids sticking to the plastic on the inside of the 1,5 mL tube.

### Notes to Protocol 6: Processing colonoids for immunostaining

28. HistoGel from Thermo Fisher Scientific comes in 10 mL aliquots and has a solid gel-like appearance at 4°C but liquefies when heated to more than 60 °C. Thaw the HistoGel at 65 °C to make 1 mL aliquots and store them at 4 °C until use.
29. It is essential to take the help of a colleague with this step. Ideally, one person pre-cools the slide by placing the slide in liquid nitrogen with long steel forceps for 20-30 seconds, while the other person pipettes the HistoGel with the colonoids onto the cooled slide. Using two P200 pipettes allows for easy handling and reduces the time required for this step. Maintaining a second P200 pipette with a higher volume is essential. Once the liquid HistoGel is added to the colonoids pellet, mixing the HistoGel with colonoids will increase this mixture’s volume. Use cut tips to keep the structure of the colonoids intact. Take good care not to create bubbles in this step as this will impact both the sectioning of the HistoGel after they are embedded in the paraffin and the images while staining. Moreover, it is important to be quick during this step and not triturate too many times as this will prematurely solidify the HistoGel.
30. While working with HistoGel, process one sample at a time from steps 3-5. Place the aliquot of HistoGel back into the thermomixer kept at 80 °C as it solidifies into a gel quickly at room temperature. HistoGel is pink color in appearance and loses color to become white and translucent after fixation.

### Notes to Protocol 7: Immunohistochemical and immunofluorescent staining of colonoids

31. Neo-Clear and absolute EtOH may be stored and reused for up to a month. Refresh reagents with new ones during high usage. For the ease of carrying out the staining, store the Neo-Clear and series of EtOH in the glass staining jars.
32. It may be difficult to locate the paraffin sections made from colonoids (embedded in HistoGel) after the antigen retrieval step in the staining procedure. Therefore, marking around the HistoGel with a waterproof permanent marker before boiling them during antigen retrieval can be useful.
33. Alternative methods of antigen retrieval may need to be adopted depending on the antibody used.
34. It is critical to cover the entire section with any solution applied in any step to avoid drying of the pellet. Normally a volume of 70-100 µL is enough to cover the pellet.
35. Be sure to use a generous volume of diluted primary antibody in order for the slides to not dry out overnight. The GelBond Film will slow down evaporation and make sure the solution does not drain away from the sections if the slides are not placed completely horizontal during this long incubation. The slides must be kept in a humid chamber inside the fridge or cold room overnight. This can be carried out by placing slides in a box containing 2-3 paper towels wet with water. As such, it is important not to let the slides dry out throughout the staining process as this will impact the visualization.
36. While placing the coverslip, it is essential not to create bubbles as this will impact the visualization and imaging process. If bubbles are created, try to remove them by carefully pushing the bubbles out of the cover glass with the applicator stick.
37. The Autofluorescence Reducing Reagent is rather volatile, so addition of more volume to the slides might be necessary.
38. Enough volume is achieved by mixing 1 + 1 drop from each of the solutions per slide to be stained.
39. Use light proof humid chamber or wrap in aluminum foil.
40. The MaxDouble M488&R650 ImmunoFluorescence Double Staining Kit has anti-fade mounting media with DAPI included (Reagent 5) which could be used.
41. The MaxFluor 488 labelled linker for mouse has an excitation wavelength of 493 nm and emission wavelength around 518 nm and will produce a green fluorescence where the mouse antibody binds. The MaxFluor 650 labelled linker for rabbit has an excitation wavelength of 652 nm and emission wavelength around 672 and will produce a red color where the rabbit antibody binds.

### Notes to Protocol 8: RNA isolation from colonoids

42. Usually, 600 µL of lysis buffer is required for lysing the colonoids. Colonoids are a bit challenging to lyse with lower volumes of the lysis buffer. Rather than processing colonoid pellets as described in Protocol 5 for RNA isolation, RNA from colonoids may also be derived by aspirating the media, rinsing with DPBS, and directly adding the RLT buffer (containing BME) to the colonoids in Matrigel in the 24-well plates. However, the steps for homogenization and removal of cell debris have to be optimized for this technique.
43. Avoid processing more than 10-12 samples at a time. Processing more samples at the same time may lead to a lower yields and quality of RNA.
44. The solution is viscous when 70% EtOH is added and less viscous when mixed, mix until viscosity is visibly reduced.
45. Discard flow-through by carefully removing the collection tube without disturbing the membrane of the spin column.
46. Avoid adding RNase free water to the side of the spin columns. The RNase free water must properly wet the membrane to obtain high yield of RNA.

### Notes to Protocol 9: Western blot protein analysis

47. NP-40 is used to break open all membranes within a cell, including the nuclear membrane.
48. NP-40 is a detergent and resuspending often creates foam. Also, the pellet is usually small making the PCV small, often between 10 and 25 µl. Buffer I do not contain NP-40 and will not create foam when resuspending.
49. After denaturing, the samples are put on ice immediately to have a better linearization of the protein. After 1 minute on ice the samples are spun down to recapture the vaporized water.
50. The run time depends on the size of the protein of interest. The smaller the protein the less running time is needed.
51. The process will generate heat which can influence the migration of the proteins. It is recommended to run the gels in a cold room or place the gel chamber in ice water if running in room temperature.
52. The dried membranes can be stored until further processing.
53. The blocking buffer can be re-used if kept away from direct light and at 4°C.
54. Tween may interfere and generate background when obtaining the images on LI-COR Odyssey. The last wash in TBS will remove tween residues and make clearer images.
55. Since phospho-STAT1 and STAT1 total have the same protein size it is important to use antibodies produced in different species and with different fluorescence tag to obtain a specific binding of the secondary antibody to the desired primary antibody and to be able to visualize both proteins on the same membrane (**Figure 4**).
56. Instead of sequential incubations, a mix of the two primary antibodies and a mix of the two secondary antibodies can be used. To be sure to avoid interference between the antibodies we prepare to incubate separately.
57. In this example GAPDH is used as housekeeping protein. There are other proteins that can fulfill the same purpose, and this must be decided in each experimental setup. The housekeeping protein should not be influenced by the stimuli in the experiment, and it needs to be of a certain size (kDa) to be compatible with the other proteins of interest. Housekeeping proteins are used to normalize the quantification of the proteins of interest.

## Results and discussion

Patient-derived colonoids are valuable pre-clinical models to study both pro-inflammatory signals and drug effects in human IECs (30–32). As proof of principle for the refined protocols presented here, we show how to investigate chemokines like CCL20 by immunohistochemistry and immunofluorescence (**Figure 2**), detection of CCL20 mRNA expression (**Figures 3A, B**) and CCL20 protein release from colonoids upon treatment with various pro-inflammatory cytokine and ligands (**Figure 3C**). It should be noted that the 3D colonoids have the basal side out. If the response of interest is manly activated from the apical site, we recommend testing published methods for apical-out cultures (33).

For studies of CCL20 mRNA expression and CCL20 protein release from colonoids, we used colonoids generated from 6 donors, 3 ulcerative colitis patients (UC) and 3 healthy controls (HC), as previously described (20). Generally, we observe large inter-individual variations in colonoid responses, including CCL20 expression. In a previous study (24), we performed series of independent experiments to examine expression of mRNA and proteins in colonoids derived from 10 different donors with various techniques (RNAseq, Western blot, immunohistochemistry, and confocal imaging). The results showed that we could reproducibly detect e.g., Major histocompatibility complex (MHC-II) expression with different techniques in a donor-dependent manner. Thus, patient-derived organoids also represent a versatile tool for studying the effects of genetic and epigenetic background to the exposure of e.g., pro-inflammatory signals, and may be used for personalized drug screening in IBD (11, 34).

There are many technical pitfalls that may influence reproducibility over time. For example, the production and use of Wnt-3A and Rspondin1 enriched media from engineered cells adds additional cell culture requirements and risks for batch-to-batch variability. Our experience is that use of Wnt-3A and Rspondin1 enriched media is a cost-effective and quite reliable approach as the activity of Wnt-3A and Rspondin1 produced *in house* appear comparable to recombinant proteins and is well maintained across different batches if we follow the protocol strictly. In a paper from 2019 (21), VanDussen, Sonnek and Stappenbech documented that conditioned medium batches from an L cell line engineered to secrete Wnt3a, R spondin 3, and Noggin (L-WRN) showed replicable batch-to-batch activity levels across multiple research teams when the protocol for generation of conditioned medium was properly executed. The authors also provide validation procedures and guidelines for quality control that may enhance reproducibility. We monitor Wnt-3A and Rspondin1 activity using TCF/LEF-luc-reporter cell line (BPS Bioscience #60501). Their activities are measured by Luciferase assay system from Promega as per the manufacturer’s instruction (Promega #E1500). If the activity in one batch is relatively higher than in others, the % of Wnt used may be diluted by increasing the volume of minigut B in differentiation medium. A recent study determined that a Wnt-3A-, R-spondin- and Nogging-free medium is optimal for colonoid differentiation (22), and this may be tested if colonoids appear less differentiated than expected. Interestingly, Wilson et al. (22) also reported that media supplemented with recombinant Wnt-3A alone did not support long-term survival of human or mouse colon organoids while Wnt-3A-conditioned media did. However, Pleguezuelos-Manzano et al. (27) recommend using synthetic Wnt agonists when growing human colon organoids from single cell. The differentiation media composition can also be fine-tuned to induce differentiation of cell types of interest (27, 29). Working mechanisms and effects, as well as applications for frequently used growth media constituents are reviewed in e.g., Holmberg et al. (29). We recommend the readers to look into publications from Mahe et al. (17), and Pleguezuelos‐Manzano et al. (27) for isolation protocols for biopsies from small intestine and variations of culturing methods.

IEO model systems are continuously refined to enhance the translational value. One aspect not discussed in the present protocols is the fact that colonic epithelium is adapted to thrive in physiological hypoxia (35). Colonoid studies are usually conducted at the atmospheric level of 20% O_2_, even though the colonic mucosal surface oxygen concentration is normally 1-2% (36). Based on evaluation studies in our lab (20, 37) we now have chosen to conduct mechanistic studies in both undifferentiated and differentiated colonoids at 2% oxygen. In general, reproducibility and impact of changes in the culturing methods may be evaluated by colonoid growth, histology and detection of cell markers for stem cells and differentiated cell (22, 27, 37).

## Data availability statement

The datasets presented in this study can be found in online repositories. The names of the repository/repositories and accession number(s) can be found below: https://www.ncbi.nlm.nih.gov/geo/, GSE172404 https://www.ncbi.nlm.nih.gov/geo/, GSE217663.

## Ethics statement

The studies involving human participants were reviewed and approved by the Central Norway Regional Committee for Medical and Health Research Ethics (reference numbers 5.2007.910, 134436 and 2013/212/REKmidt). The patients/participants provided their written informed consent to participate in this study.

## Author contributions

TB and AS supervised the study. SG, IB, MH, HK, AG, and TB contributed to experimental design, generated, and analyzed data. AS collected and characterized patient samples. SG, IB, MH, HK, and TB made figure panels. SG, IB, MH, and TB drafted the manuscript. All authors contributed to the article and approved the submitted version.
