# Supplementary material for: Comprehensive protocols for culturing and molecular biological analysis of IBD patient-derived colon epithelial organoids
Source: Front Immunol. 2023 Feb 23;14:1097383. doi: 10.3389/fimmu.2023.1097383 (PMC9995983; doi:10.3389/fimmu.2023.1097383)
Supplement: Supplementary file 1 [file DataSheet_1.pdf]

Supplementary Material

Comprehensive protocols for culturing and molecular biological analysis of IBD patient-derived colon epithelial organoids

Shreya Gopalakrishnan [1], Ingunn Bakke [1,2], Marianne Doré Hansen[1,2], Helene Kolstad Skovdahl [1,3], Atle van Beelen Granlund [1,3,4], Arne Kristian Sandvik [1,3,4], and Torunn Bruland [1,4,5*]

1) Department of Clinical and Molecular Medicine (IKOM), Faculty of Medicine and Health Sciences, NTNU- Norwegian University of Science and Technology, Trondheim, Norway.

2) Clinic of Laboratory Medicine, St. Olav's University Hospital, Trondheim, Norway

3) Centre of Molecular Inflammation Research (CEMIR), Faculty of Medicine and Health Sciences, NTNU- Norwegian University of Science and Technology, Trondheim, Norway.

4) Department of Gastroenterology and Hepatology, Clinic of Medicine, St. Olav's University Hospital, Trondheim, Norway.

5) Clinic of Medicine, St. Olav's University Hospital, Trondheim, Norway.

***Corresponding authors contact information**

Torunn Bruland

NTNU Department of Clinical and Molecular Medicine, NO-7491 Trondheim, Norway

Email: torunn.bruland@ntnu.no

Supplementary protocol to Protocol 10: ELISA to examine CCL20 release in conditioned media from colonoids.

Kits from R&D systems use solid-phase Sandwich ELISA and manufacturer's instruction for the kit was followed with minor modifications as described below. Kit used: CCL20 (DY360). Example of CCL20 release to the conditioned media upon treatment with IBD-relevant is shown in **Figure 3B** (1). The kit works sufficiently with half of the volume of what is recommended by the manufacturer to detect CCL20 (*see* notes 1-4).

Protocol SP1:

1. Prepare working concentration of capture antibody in DPBS at room temperature as per concentrations mentioned in the certificate of analysis. Add capture antibody in DPBS to multi-channel pipette reservoir. Pipette 50 µL per well to the side of the wells using a multi-channel P200 pipette. Tap on the sides of the plate to make sure all the wells are covered with the liquid (*see* notes 1-4).
2. Seal the plate with adhesive sealing tape and incubate overnight at room temperature in the dark.
3. The following day thaw the conditioned media samples collected from the colonoids on ice (*see* note 5).
4. While the media is thawing, wash the plate by removing the seal, aspirating the DPBS containing capture antibody and adding 300 µL wash buffer per well. Wait for 5 seconds and aspirate the wash buffer completely (*see* note 6). This step is performed with a HydroSpeed 96-well plate washer that is programmed to do three washes, with 4 seconds aspiration time per wash. Remove excess liquid (if any) in the plate by inverting and tapping the plate on a paper towel. Follow these steps for all the washes mentioned in the procedure.
5. Block the plate by adding 150 µL of reagent diluent per well to the side of the well and incubate for 1 hour at room temperature. During the incubation, prepare the samples and standard required for the next step.
6. We recommend using the following calculation for 1-2 plates. To prepare standard, label eight 1.5 mL microfuge tubes with alphabets A-H. Add 1000 µL of reagent diluent to tube A

and 500 µL of reagent diluent to tubes B-H. Prepare working concentration of the top standard (see the certificate of analysis of the kit and *see* note 44) in tube A per 1000 µL, mix thoroughly and perform a two-fold serial dilution by transferring 500 µL from tube A to tube B. Mix thoroughly, and transfer 500 µL from tube B to C, mix thoroughly and transfer 500 µL from C to tube D. Repeat this until you have 1 mL in tube H.

7. Prepare undiluted, 1:10 and 1:100 dilutions of conditioned media in the reagent diluent (*see* note 7).
8. Remove seal carefully, aspirate and wash the plate as per step 4 and remove excess liquid by inverting and tapping the plate on paper towels.
9. Add 50 µL of standard and samples in duplicates to the side of the well (*see* note 8). Seal the plate and incubate for 2 hours at room temperature.
10. Prepare the detection antibody in reagent diluent in 15 mL tube as per the working concentration mentioned in the certificate of analysis. Remove seal from plate carefully, aspirate and wash the plate as per step 4. Remove excess liquid by inverting and tapping the plate on paper towels.
11. Add detection antibody in reagent diluent to multi-channel pipette reservoir and pipette 50 µL per well to the side of the wells using a multi-channel P200 pipette and incubate for 2 hours at room temperature.
12. Prepare working concentration of streptavidin-HRP in reagent diluent in 15 mL tubes as per dilution mentioned in the certificate of analysis of the kit. Cover the 15 mL tube in aluminium foil to protect from light. Remove seal carefully, aspirate and wash the plate as per step 4 and remove excess liquid by inverting and tapping the plate on paper towels.
13. Add 50 µL of streptavidin-HRP in reagent diluent per well to the side of the wells. Seal and cover plate with aluminium foil or place plate in the dark and incubate for 20 minutes.
14. Remove seal carefully, aspirate liquid, wash the plate as per step 4, and remove excess liquid by inverting and tapping the plate on paper towels.
15. This step has to be carried out inside a fume hood as the TMB substrate solution B is toxic. Take a 15 mL tube, cover with aluminium foil and prepare substrate solution by mixing equal volumes of TMB substrate solution A and B (3 mL of each solution per plate). Add to multi-channel pipette reservoir and pipette 50 µL per well of substrate solution mix to the side of the wells using a multi-channel P200 pipette, seal the plate and cover with aluminium foil. Incubate in the dark for 20 minutes.

16. Take the required volume of stop solution (3 mL per plate) in a multi-channel pipette reservoir. Using a multi-channel P200 pipette, add 25 µL per well to the side of the well. Seal the plate and tap the four sides of the plate carefully for the stop solution to be suspended evenly in the substrate solution. The reaction will be represented by the change of blue colour of the substrate, to yellow colour (*see* note 8).
17. Measure the optical density of the wells by reading the plate in a microplate reader that is set to 450 nm, with a wavelength correction set to 570 nm.

Notes and troubleshooting: ELISA to examine CCL20 release in conditioned media from colonoids.

1. Prepare stock aliquots of capture antibody (in DPBS), detection antibody and standards (in reagent diluent), as per the stock concentration mentioned in the certificate of analysis sheet and freeze them until use. These are aliquoted are prepared based on volumes required for per plate.
2. Throughout the process, it is important to use calibrated pipettes without errors as this can impact the readings to a great extent. Bubbles while pipetting should be avoided as this could potentially vary the sample volumes and impact the assay. Thorough mixing of antibodies, standards/conditioned media or Streptavidin HRP, in DPBS or reagent diluent is essential. Do not allow plates to dry out completely. Pipette to the sides of the well and not directly to the bottom of the well during the entire procedure.
3. Prepare the wash buffers and reagent diluent on the day of capture antibody addition. Unsterile and minor particles in the reagent diluent can interfere with assay results, so filtration of the reagent diluent is essential.
4. The assay requires good quality of BSA. An example of this is #A7906-500G from Sigma.
5. Thawing supernatant on ice can take a long time. To speed up the process, you can place the tubes horizontally on ice.
6. If a washing machine is not available, you may aspirate the DPBS containing capture antibody from the plate and add wash buffer with multi-channel P200 pipette, wait for four seconds and aspirate the wash buffer. Repeat this step three times. However, it is important to be quick to avoid drying out the wells. Further, in the third wash step, complete removal of wash buffer is required for efficient results which may be better achieved with a washing machine than manually adding the wash buffer and drying plates.

7. CCL20 is secreted in very high amounts upon stimulation of colonoids with TNF or Poly(I:C). Therefore, one may need to optimize dilutions for detecting this chemokine depending on the assay performed.
8. It is important to have duplicates or triplicates of each sample and the standards. Use a 96-well U-well plate to prepare the different dilutions of samples. Although 50 µL volume of sample is required per well, prepare for 60 µL volume per well. Quick spin the plate in a centrifuge before reading the optical density if the substrate solution or stop solution droplets are present on the side of the wells of the plate. The presence of droplets on the sides of the wells can impact the optical density reading.

Reference

1. Skovdahl HK, Granlund A, Ostvik AE, Bruland T, Bakke I, Torp SH, et al. Expression of CCL20 and Its Corresponding Receptor CCR6 Is Enhanced in Active Inflammatory Bowel Disease, and TLR3 Mediates CCL20 Expression in Colonic Epithelial Cells. PLoS One. 2015;10(11):e0141710.
